# Supplementary material for: Gene-environment correlation: the role of family environment in academic development
Source: Mol Psychiatry. 2024 Sep 4;30(3):999–1008. doi: 10.1038/s41380-024-02716-0 (PMC11835719; doi:10.1038/s41380-024-02716-0)
Supplement: Supplementary file 1 — Supplementary Notes and Figures [file 41380_2024_2716_MOESM1_ESM.pdf]

Supplementary Material

Gene-environment correlation: The role of family environment in academic development

Quan Zhou[1], Agnieszka Gidziela[1], Andrea Allegrini[2,3], Rosa Cheesman[2,4], Jasmin Wertz[5], Robert Plomin[2], Kaili Rimfeld[2,6], & Margherita Malanchini[1,2]

[1]School of Biological and Behavioural Sciences, Queen Mary University of London
[2]Social, Genetic & Developmental Psychiatry Centre, Institute of Psychiatry, Psychology & Neuroscience, King's College London
[3]Division of Psychology and Language Sciences, University College London
[4]PROMENTA Research Center, Department of Psychology, University of Oslo
[5]School of Philosophy, Psychology and Language Sciences, University of Edinburgh
[6]Department of Psychology, Royal Holloway, University of London

Supplementary Notes

Note 1. Deviation from the preregistered analyses.

Note 2. Background information of neighborhood measures.

Note 3. Creation of Stimulating home environment and TV consumption scales.

Note 4. Details of exploratory and confirmatory factor analysis.

Note 5. Description of mediation models.

Supplementary Figures

Figure 1. Correlations between environmental pollution measures.

Figure 2. Correlations between neighbourhood quality measures.

Figure 3. Correlations between neighbourhood economy measures.

Figure 4. Scree plots of environmental pollution, neighbourhood quality and neighbourhood economy measures.

Figure 5. Factor structure of environmental pollution measures.

Figure 6. Factor structure of neighbourhood quality measures.

Figure 7. Factor structure of neighbourhood economy measures.

Figure 8. Correlations between parent and self-rated home environment items at age 9.

Figure 9. Scree plots of parent and self-rated home environment items at age 9.

Figure 10. Factor structure of parent and self-rated home environment items at age 9.

Figure 11. Distributions of cross-sectional composites.

Figure 12. Distributions of square root transformed cross-sectional composites.

Figure 13. Correlations between untransformed and square root transformed cross-sectional composites.

Figure 14. Correlations between parent-rated environmental variables at age 7.

Figure 15. Correlations between parent and self-rated environmental variables at age 9.

Figure 16. Correlations between parent and self-rated environmental variables at age 12.

Figure 17. Correlations between self-rated environmental variables at age 16.

Figure 18. Scree plots of parent and self-rated environmental variables at first contact and ages 7, 9, 12 and 16.

Figure 19. Factor structure of parent-rated environmental variables at age 7.

Figure 20. Factor structure of parent and self-rated environmental variables at age 9.

Figure 21. Factor structure of parent and self-rated environmental variables at age 12.

Figure 22. Factor structure of self-rated environmental variables at age 16.

Figure 23. CFA models of parent and self-rated latent cross-sectional composites at ages 9 and 12.

Figure 24. Cross-sectional composites.

Figure 25. Correlations between composites.

Figure 26. Cognitive and noncognitive PGS effects onacademic achievement over development mediated by neighbourhood environments.

Figure 27. Educational attainment, cognitive and noncognitive PGS effects on academic achievement over development mediated by individual measures of the family environment.

Figure 28. Environmental mediation models using other cognitive and noncognitive polygenic scores. Cognitive and noncognitive PGS effects on academic achievementover development mediated by family environmental composites.

Figure 29. Environmental mediation models using other cognitive and noncognitive polygenic scores. Cognitive and noncognitive PGS effects on academic achievement over development mediated by individual measures of the family environment.

Figure 30. Indirect (environmentally mediated) educational attainment PGSeffects on academic achievement before (pink) and after (blue) accounting for SES using two-mediators models.

Figure 31. Indirect (environmentally mediated) cognitive PGS effects on academic achievement before (pink) and after (blue) accounting for SES using two-mediators models.

Figure 32. Indirect (environmentally mediated) noncognitive PGS effects on academic achievement before (pink) and after (blue) accounting for SES using two-mediators model.

Figure 33. Environmentally mediated cognitive PGS effects on academic achievement across development, separated into within and between family effects.

Figure 34. Environmentally mediated noncognitive PGS effects on academic achievement across development, separated into within and between family effects.

Supplementary Note 1. Deviation from the preregistered analyses.

This study includes all the developmental, cross-sectional analyses preregistered at the following link: https://osf.io/tyf4v/. We are currently working on a follow-up study that extends our analyses to consider longitudinal mediators, therefore modelling stability and change in the environmental mediators.

Supplementary Note 2. Background information of neighborhood measures.

Of the 10469 families with recorded postcodes in both 1998 and 2005, 55% maintained the same address. This consistency persisted, with 49% of the 9803 families retaining the same postcode from 1998 to 2010. Between 2005 and 2010, 86% of the 10121 families had unchanged postcodes, underscoring the relative residential stability of the cohort. More details can be found in the TEDS data dictionary: https://www.teds.ac.uk/datadictionary/studies/measures/postcode_linked_data.htm

Supplementary Note 3. Creation of Stimulating home environment and TV consumption scales.

In order to create the age 9 scales capturing variation in the home environment, we first explored the factor structure of parent and self-reported data separately. Example items related to the home environment included: '*The TV is on when the child is doing homework*', '*I discuss school activities with the child*' and '*Hours of TV watched per weekend day*'. Items were scored on a six-point Likert scale (0= 0 hours, 1= 1 hour up to 5 = 5 or more hours) with higher score indicating more enriching home environment. Correlations between parent and self-reported home environment items are illustrated in **Supplementary Figure 8**.

To explore the factor structure of the home environment items at age 9, we conducted an exploratory factor analysis (EFA) of parent and self-rated items. EFA analyses were conducted in *psych* for R (1, 2) and involved a sample of 3238 and 2941 independent twins, for parent and self-rated data respectively, created by randomly selecting one twin per pair. Results of the EFA are presented in **Supplementary Figure 9** and **Supplementary Figure 10**.

We adopted the data-driven approach and created parent-rated *Stimulating home environment* and self-rated *TV consumption* scales. The parent-rated *Stimulating home environment* scale was constructed as standardised mean of parent-rated items *How many books, Child taken to museum in the past year* and *Computer at home used by child.* The self-rated *TV consumption* scale was calculated as the standardised mean of self-rated items *Hours of TV watched per school day* and *Hours of TV watched per weekend day*, as indicated by the EFA. Although the parent-rated variable showed low internal validity, the self-rated scales showed good internal validity (Cronbach's α= 0.38 for parent-rated and 0.67 for self-rated scales).

Supplementary Note 4. Details of exploratory and confirmatory factor analysis.

We examined model fit indices, including the Comparative Fit Index, Tucker-Lewis Index, Akaike Information Criterion, Bayesian Information Criterion and Root Mean Square Error of Approximation to determine the goodness of fit of each model.

EFA analyses were conducted using a sample of up to 11,497 randomly selected unrelated twins and CFA models were tested on the other half of the randomly selected sample (the other randomly selected sibling). Correlation matrices between environmental variables at each age are presented in Supplementary Figures 14-17, scree plots are presented in Supplementary Figure 18 and factor structures yielded by each EFA are illustrated in Supplementary Figures 19-22.

CFA models are illustrated in Supplementary Figure 23 and model fit indices are presented in Supplementary Table 1. Due to the fact that successful convergence of CFA models requires >2 manifest variables to define the latent composite, standardized mean scores were derived for latent constructs comprising <3 manifest variables and for those that showed convergence problems or poor model fit. All cross-sectional composites, along with methods used for their construction, are illustrated in Supplementary Figure 24. Correlations between the cross-sectional composites are presented in Supplementary Figures 25.

Supplementary Note 5. Description of mediation models.

The SEM for this mediation model for the *i* th subject ($1 \leqslant i \leqslant n$) is given by:

$$z_i = \beta_0 + \beta_{xz} x_i + \varepsilon_{zi},$$
$$y_i = \gamma_0 + \gamma_{zy} z_i + \gamma_{xy} x_i + \varepsilon_{yi}$$

It is posited that the error terms (ϵz_i, ϵy_i) are uncorrelated, a critical presumption for causal inference when conducting mediation analysis. The assumption of multivariate normality for the error terms is also made, as it is an essential precondition for defining direct, indirect, and total effects. It should be highlighted that the two structural equations are interconnected, and the inference drawn from them is concurrent, rather than from two separate standard regression equations. More information can be found here (3).

Population-level mediation analyses

We conducted mediation analyses (Baron & Kenny, 1986; Preacher & Kelley, 2011) using the lavaan package for R to examine the direct and indirect effects of the prediction from

genetic predisposition (quantified as the PGSs of educational attainment, cognitive and noncognitive skills) and manifestation of variation in academic achievement (Figure 1).

The mediation model estimates the indirect effect of the predictor (X) on the outcome (Y) via a mediator, i.e., an intervening variable (mediator; M; in this project, the single-timepoint or developmental environmental composite) by regressing M on X and regressing Y on both X and M using two separate equations:

$$1)\ M_i = d_{M.X} + aX_i + e_{M.Xi}$$

Where Mi is the mediator for individual I; dM.X is the intercept for the mediator (M); aXi is the slope of M regressed on the predictor (X) and eM.Xi is the measurement error for individual i.

$$2)\ Y_i = d_{Y.MX} + bM_i + c'X_i + e_{Y.MXi}$$

Where Yi is the outcome for individual I; dY.MX is the intercept for the outcome (Y); bMi is the slope of the outcome (Y) regressed on the mediator (M) controlling for the predictor (X); c'Xi is the slope of the outcome (Y) regressed on the predictor (X) controlling for the mediator (M) and eY.MX is the measurement error for individual i.

The indirect effect of the predictor on the outcome (i.e., the mediation effect) is defined by a^b^, with the sample estimate signified by the circumflex ("^").
When $a^{\wedge} \times b^{\wedge} = c^{\wedge} - c^{\wedge\prime}$, then $c^{\wedge} = a^{\wedge} \times b^{\wedge} + c^{\wedge\prime}$. Implementing SEM allows for, a^ and b^ can be derived simultaneously and for testing more complex models with latent class predictor, outcomes, and mediators.

We applied a sandwich correction to account for non-independence of observation (i.e. relatedness).

We ran 2 types of population-based mediation models:

a) Population-based mediation models using cross-sectional environmental mediators

b) Population-based mediation models using longitudinal environmental mediators

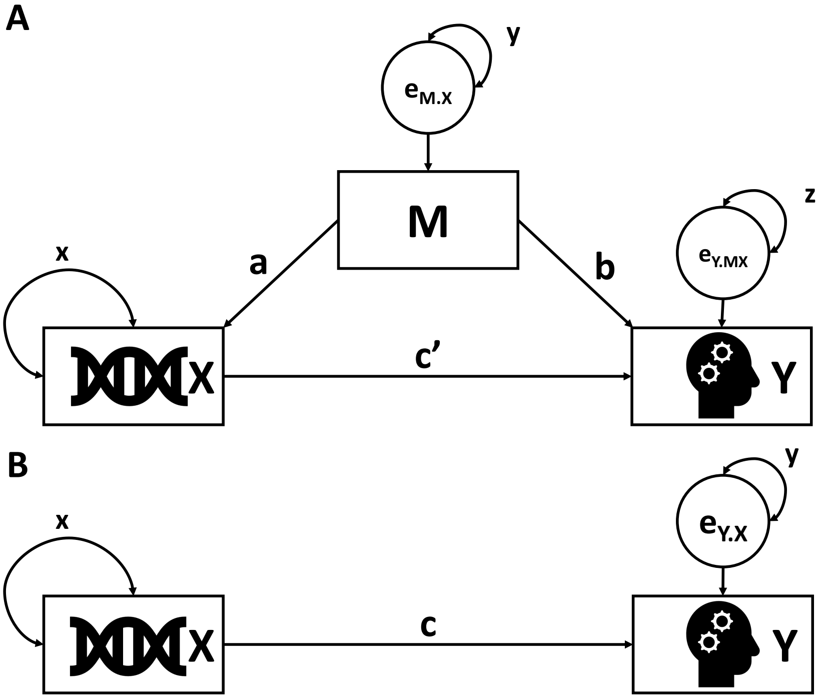

Note: Mediation model of the DNA (X) of cognitive/educational outcomes (Y) that is (panel A) versus is not (panel B) mediated by M, which denotes the multi-environmental composite, either single-timepoint or developmental. Circles indicate residuals. Parameters a, b and c represent regression weights. Parameters x, y and z represent variance parameters.

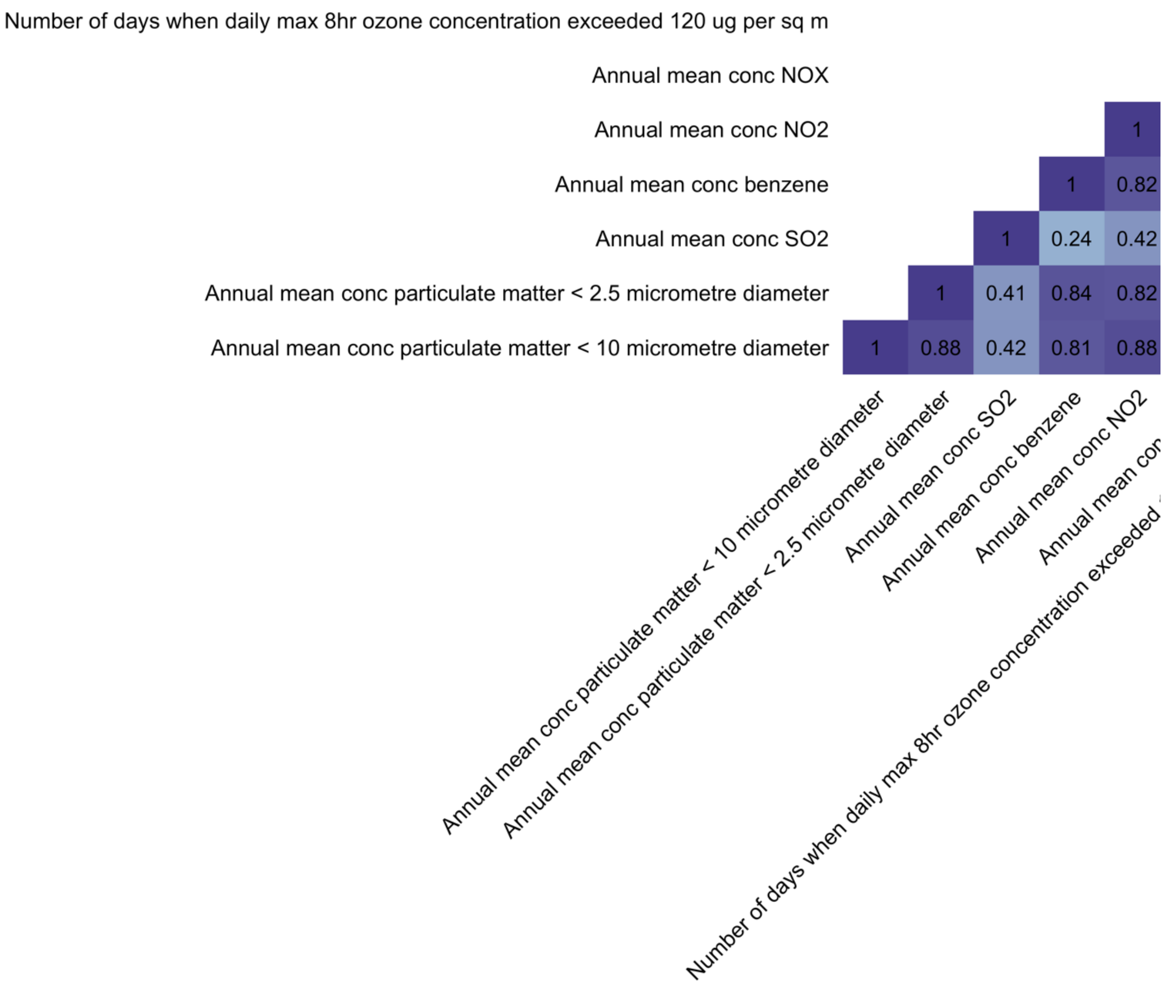

Supplementary Figure 1. Correlations between environmental pollution measures.

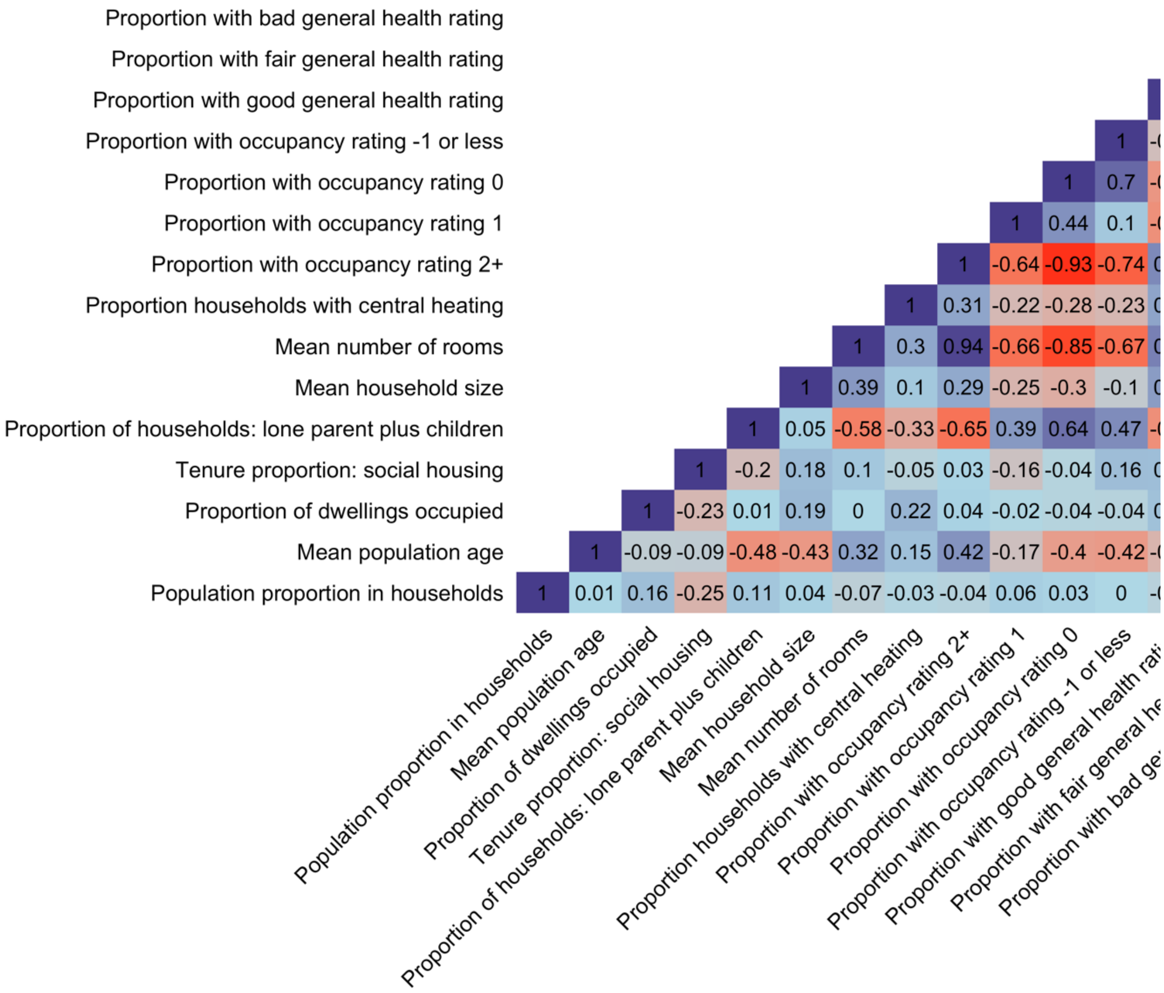

Supplementary Figure 2. Correlations between neighbourhood quality measures.

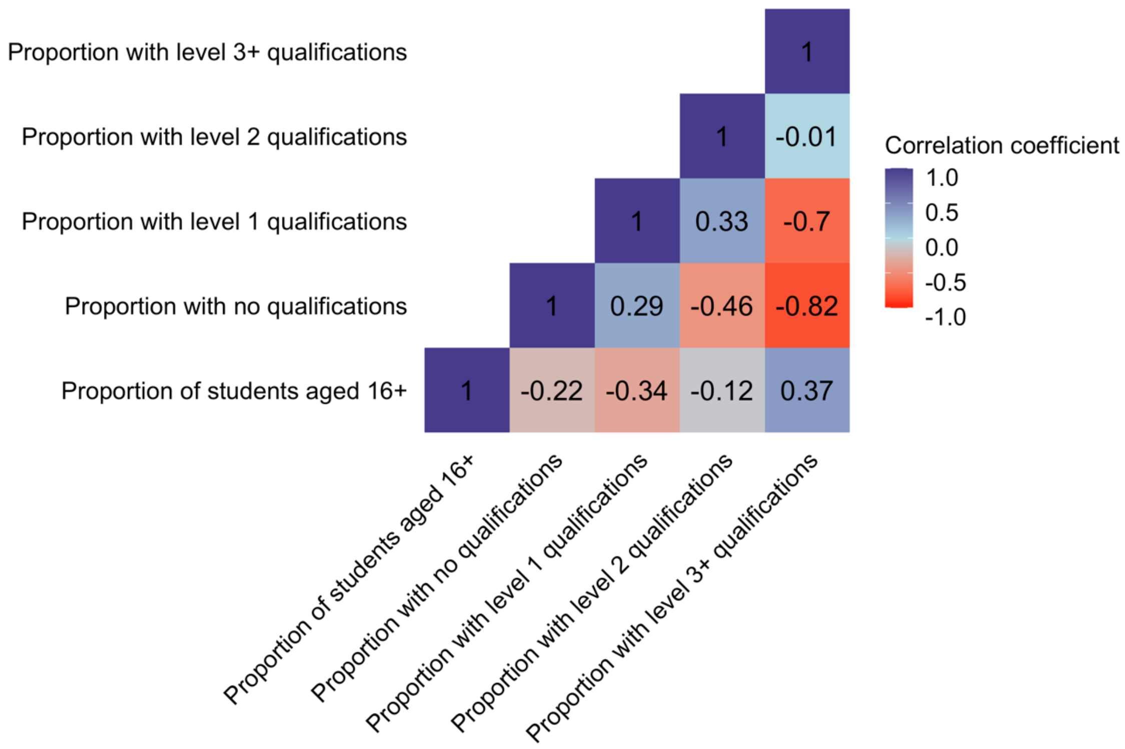

Supplementary Figure 3. Correlations between neighbourhood economy measures.

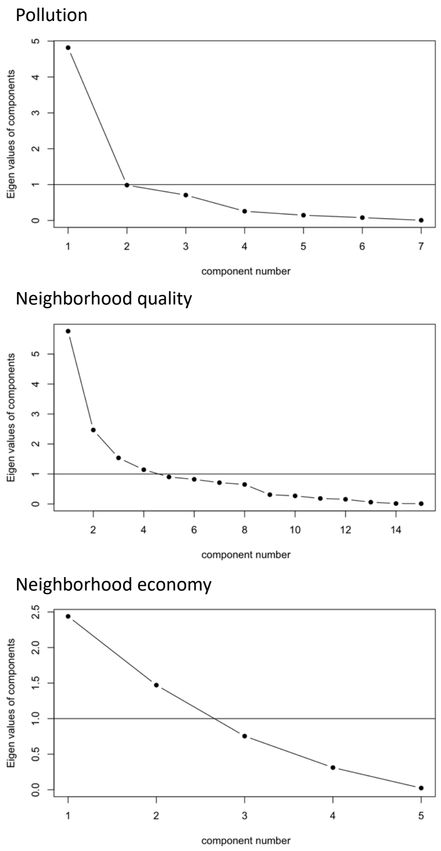

Supplementary Figure 4. Scree plots of environmental pollution, neighbourhood quality and neighbourhood economy measures.

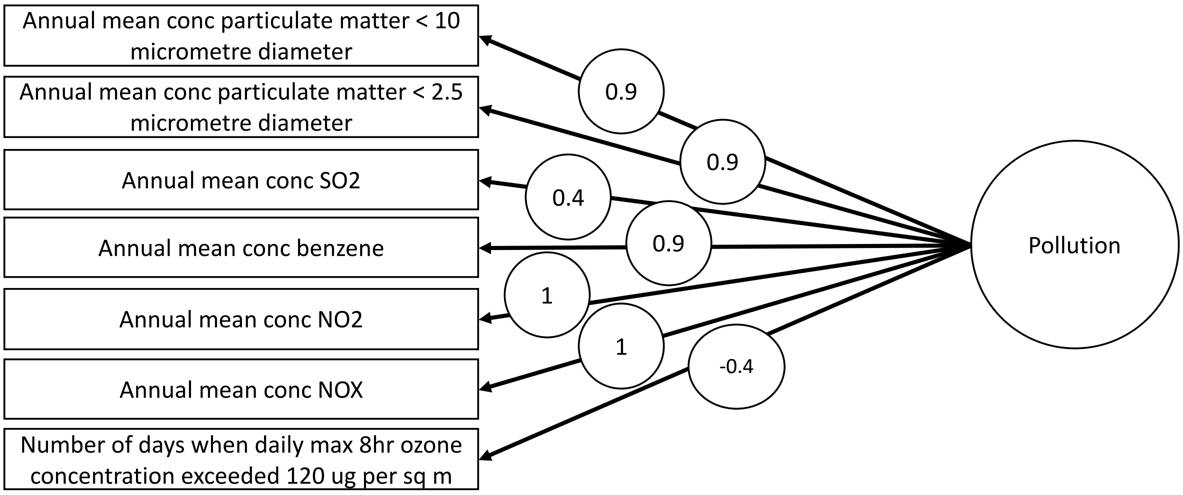

Supplementary Figure 5. Factor structure of environmental pollution measures.

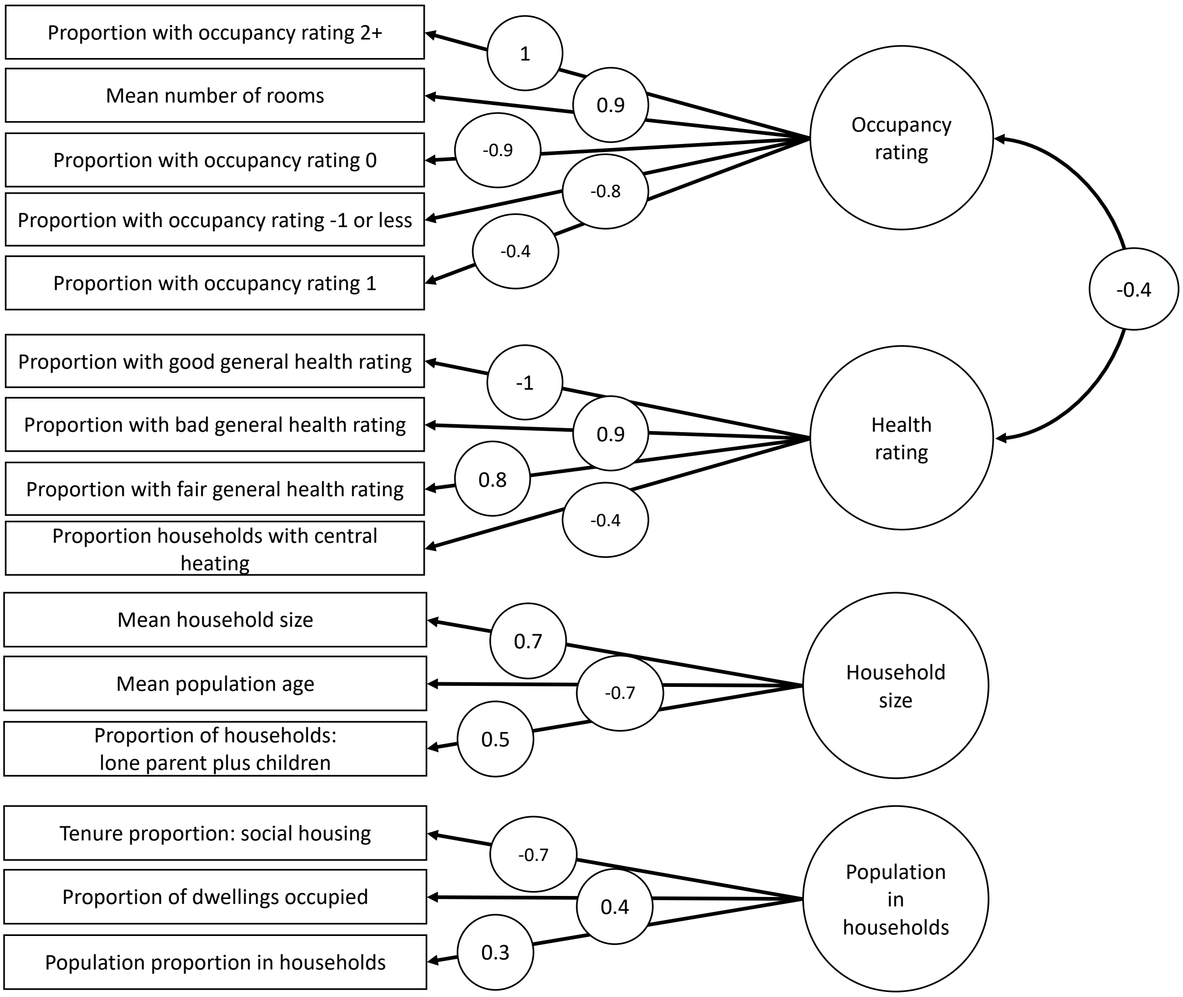

Supplementary Figure 6. Factor structure of neighbourhood quality measures.

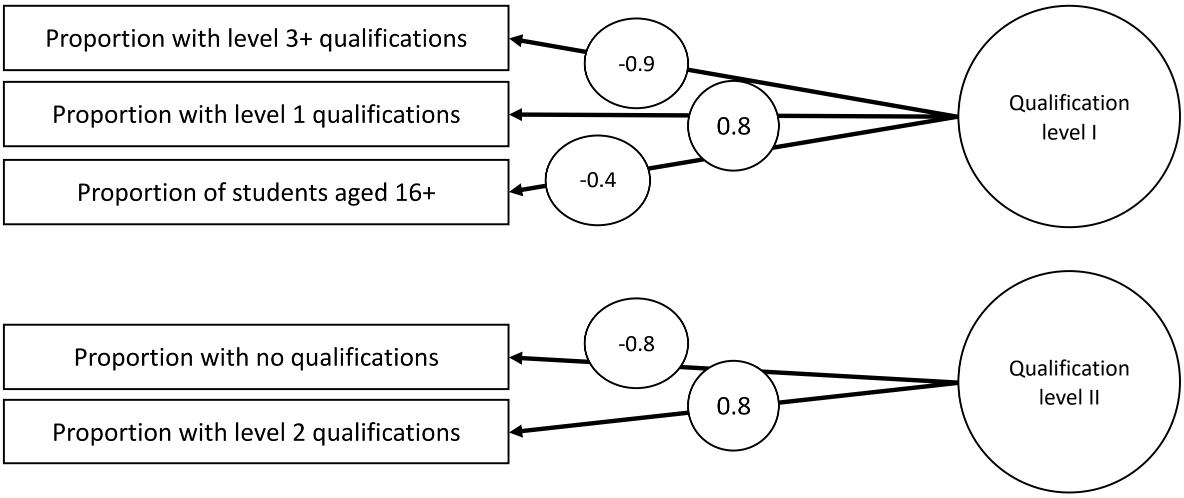

Note. Due to no theoretical coherence of qualification level II factor, only qualification level I factor was used in mediation analyses.

Supplementary Figure 7. Factor structure of neighbourhood economy measures.

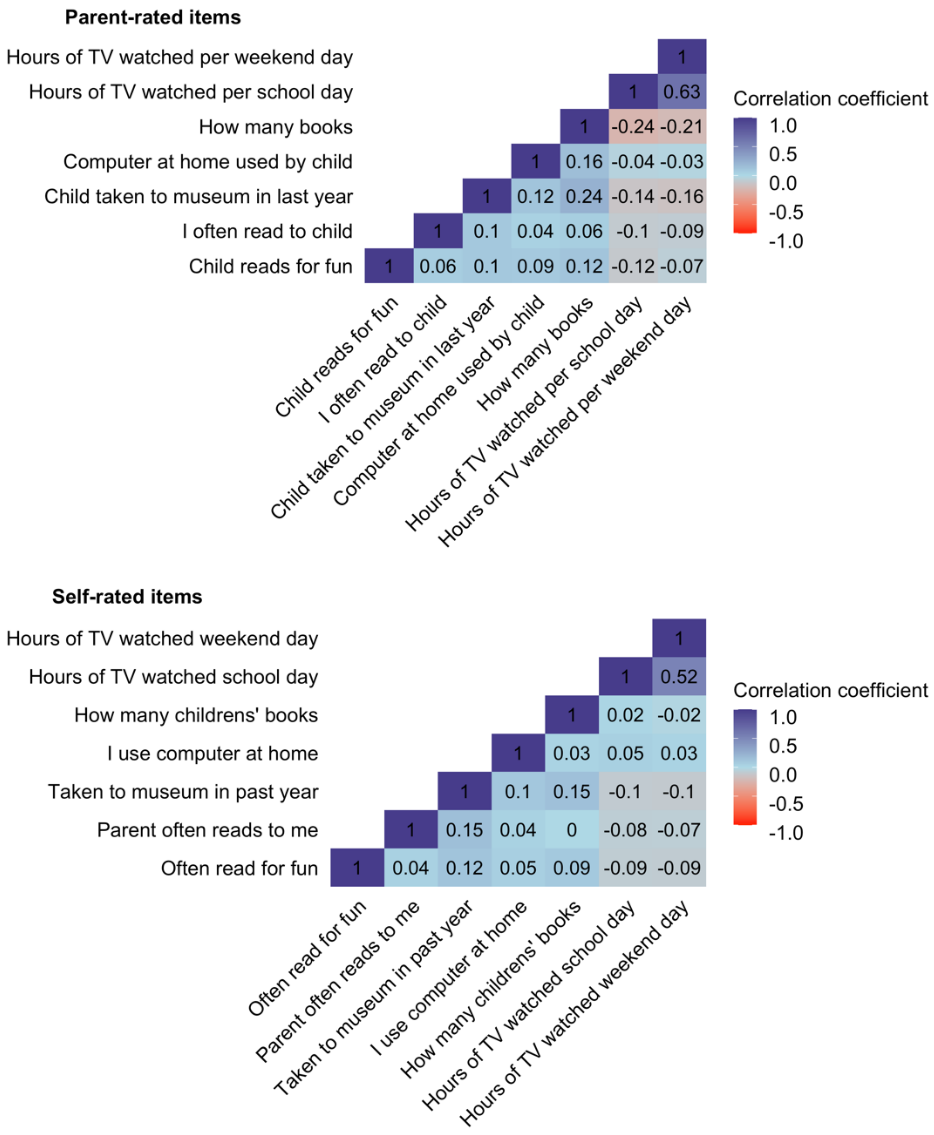

Supplementary Figure 8. Correlations between parent and self-rated home environment items at age 9.

Parent-rated items

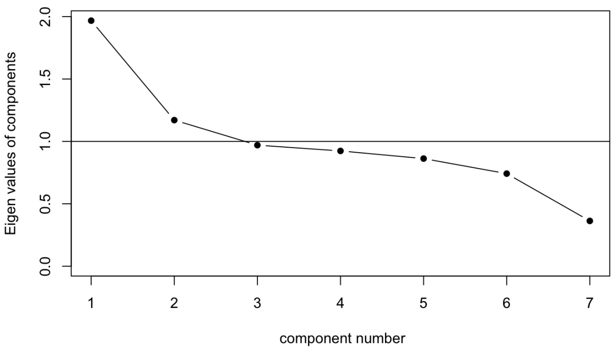

Self-rated items

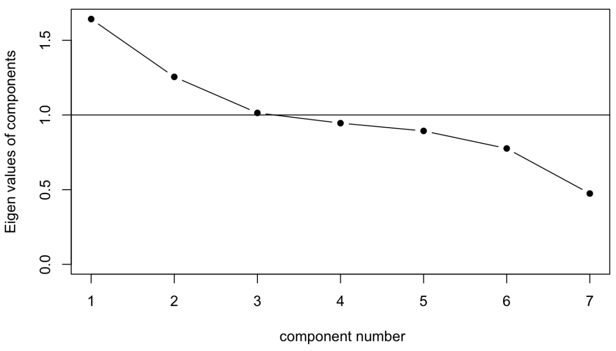

Supplementary Figure 9. Scree plots of parent and self-rated home environment items at age 9.

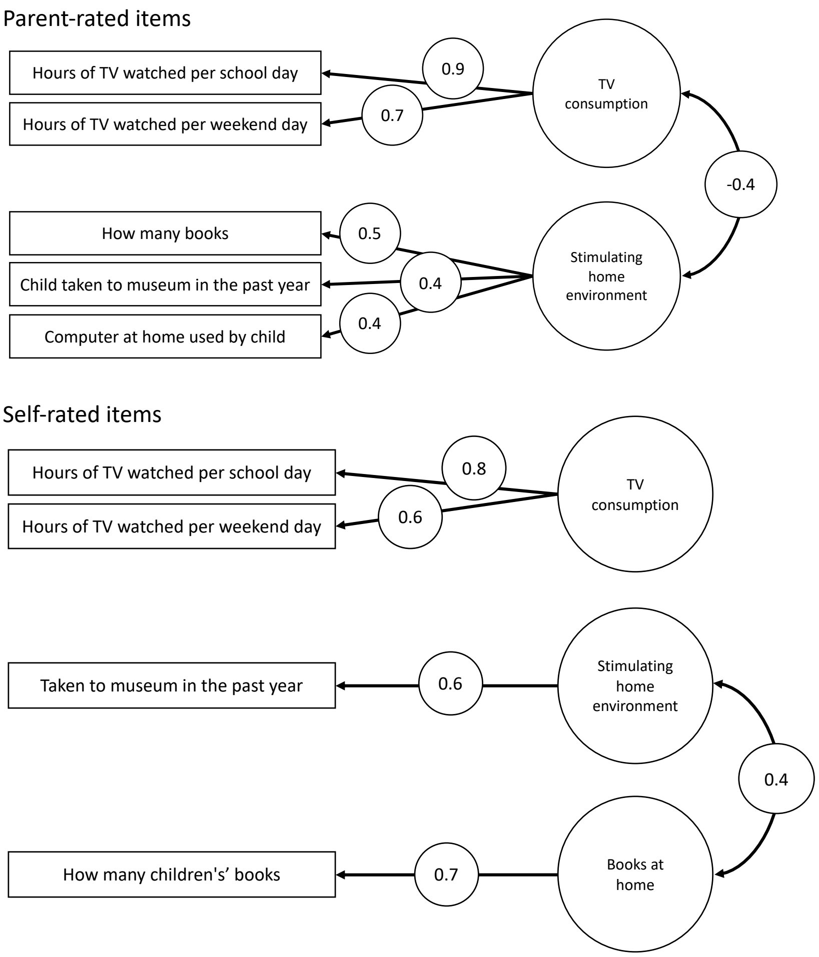

Supplementary Figure 10. Factor structure of parent and self-rated home environment items at age 9.

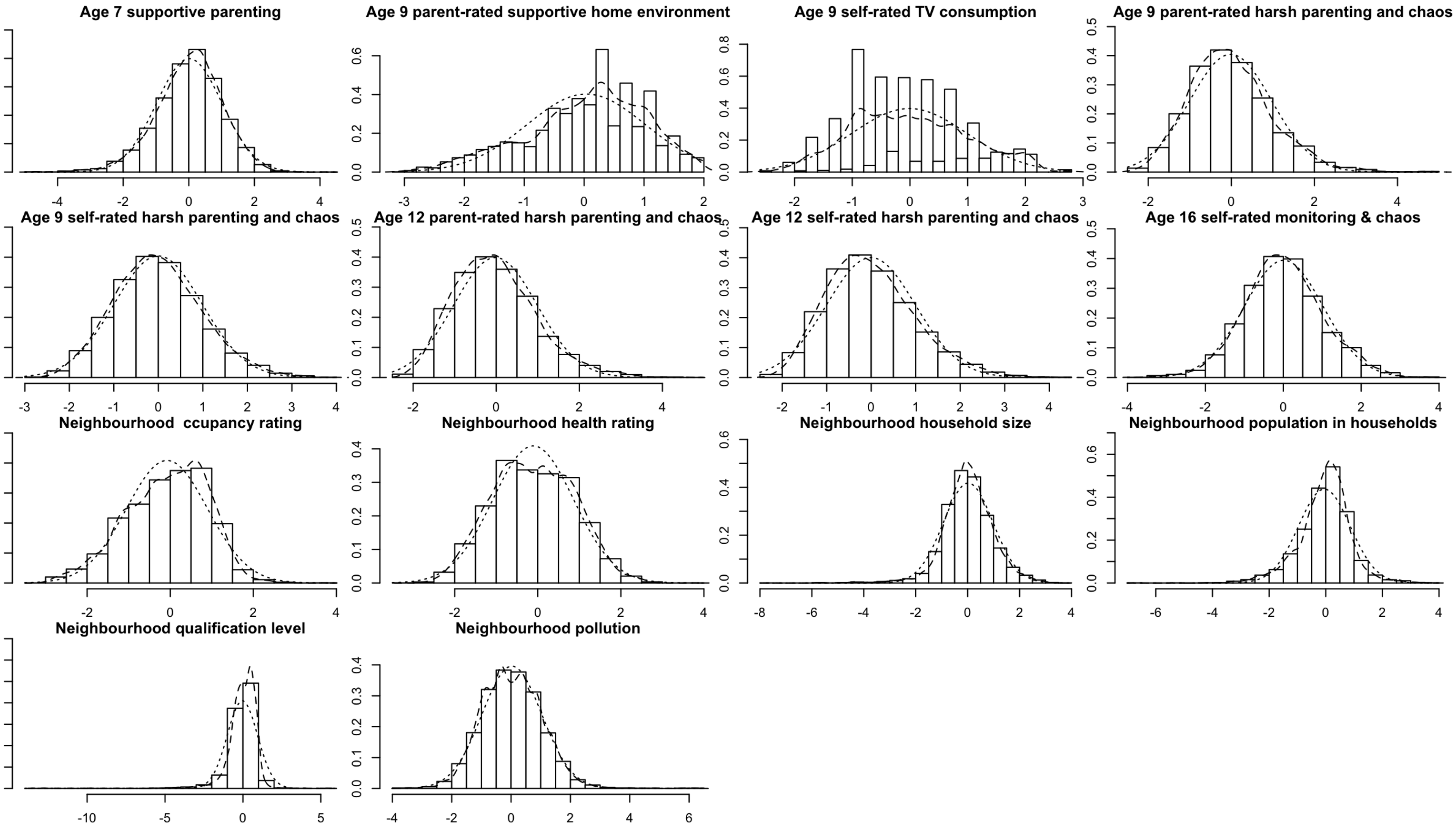

Supplementary Figure 11. Distributions of cross-sectional composites.

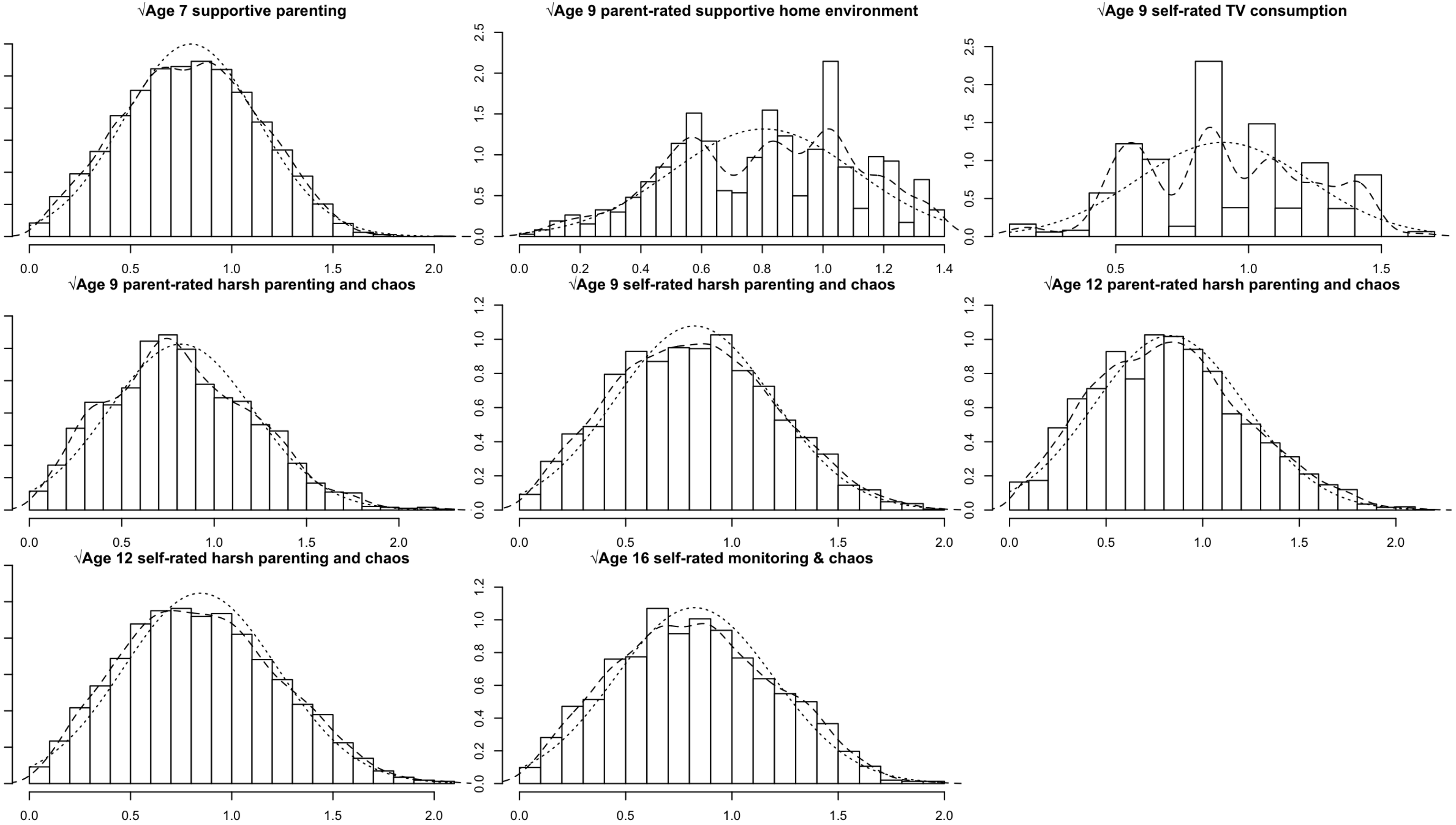

Supplementary Figure 12. Distributions of square root transformed cross-sectional composites.

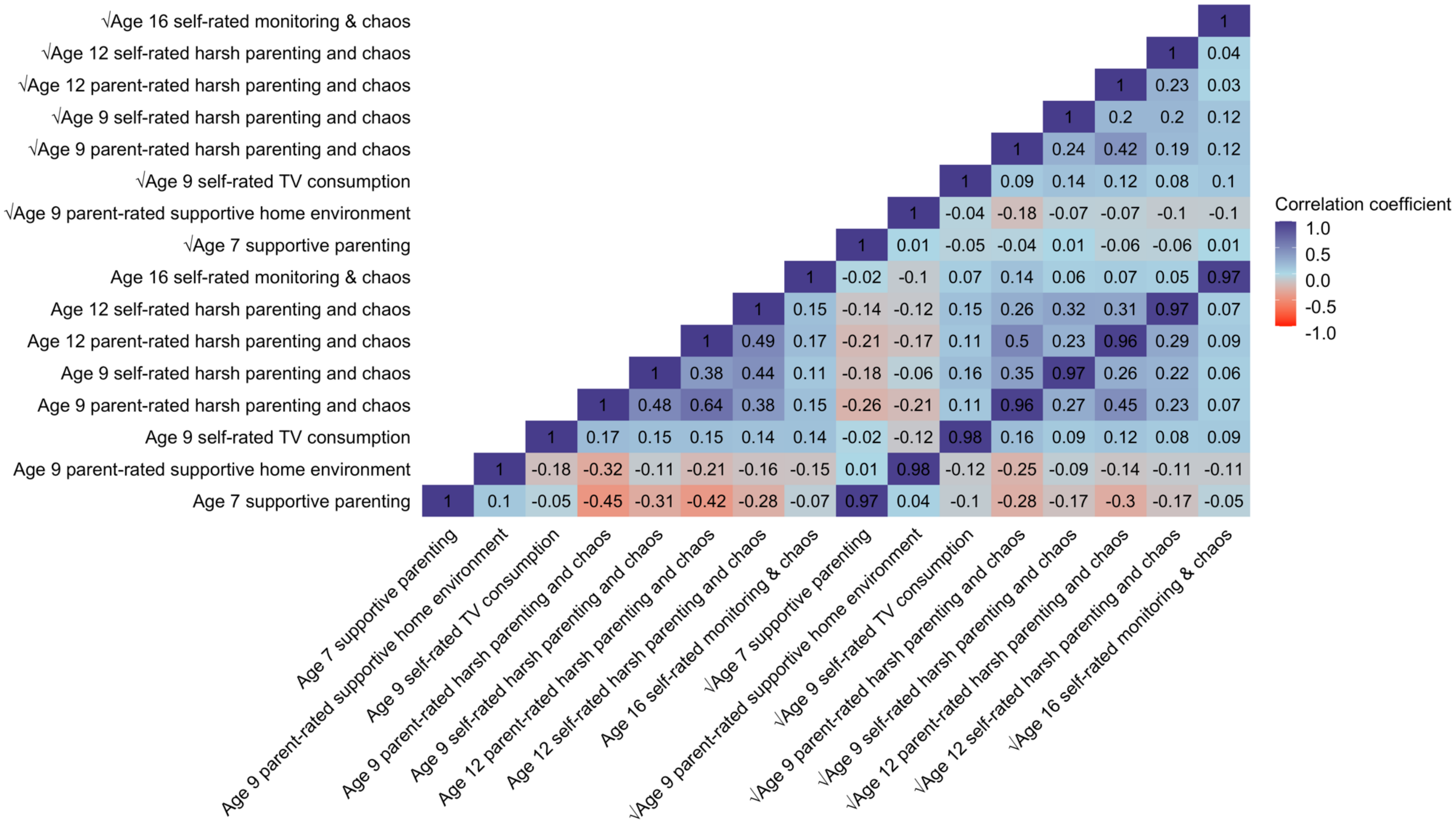

Supplementary Figure 13. Correlations between untransformed and square root transformed cross-sectional composites.

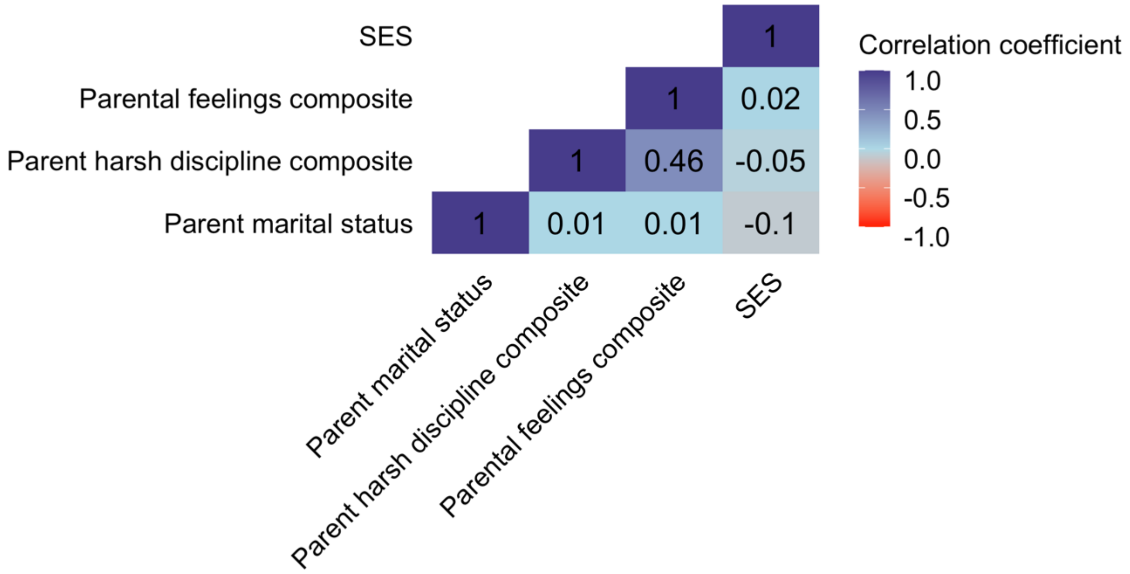

Supplementary Figure 14. Correlations between parent-rated environmental variables at age 7.

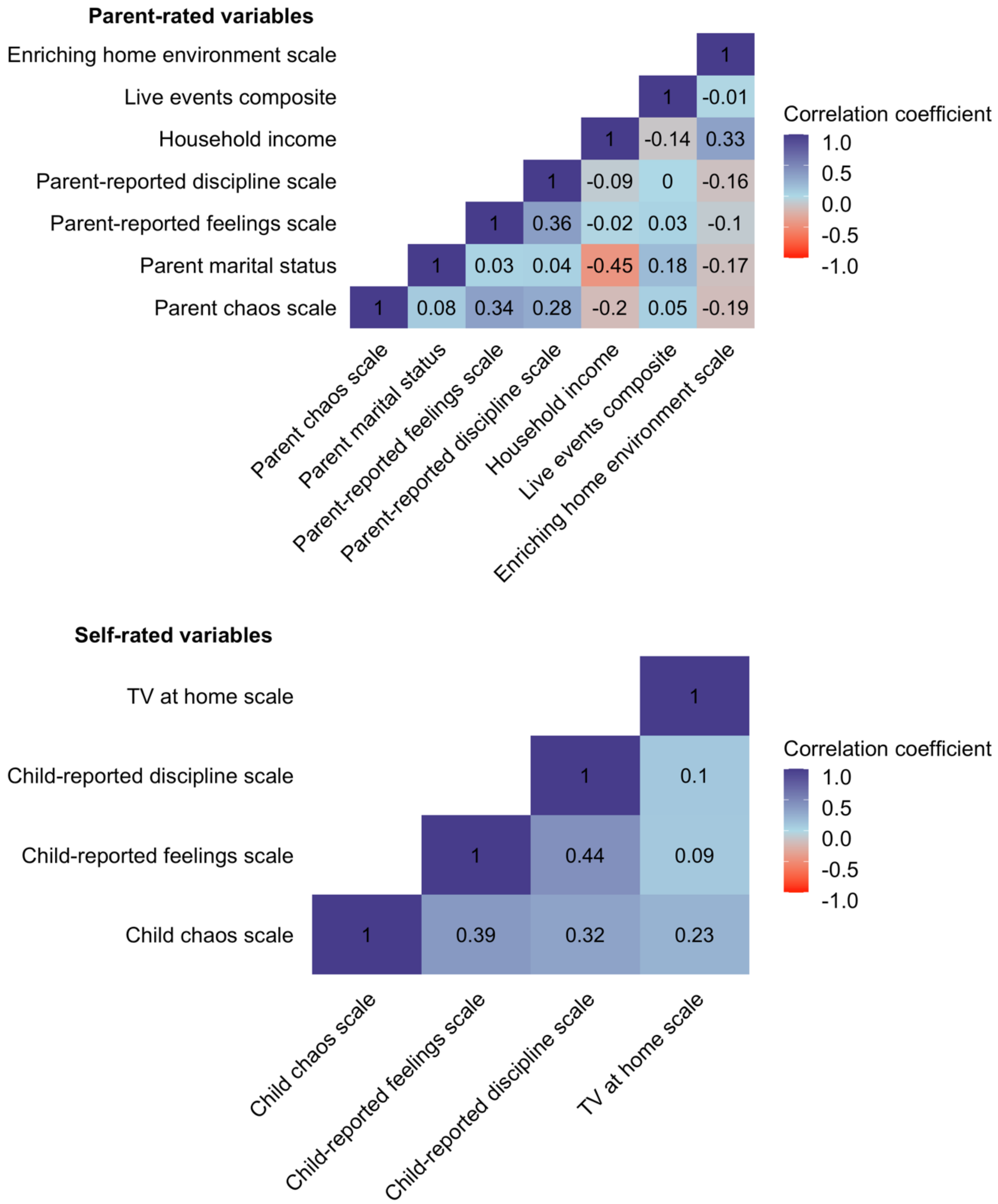

Supplementary Figure 15. Correlations between parent and self-rated environmental variables at age 9.

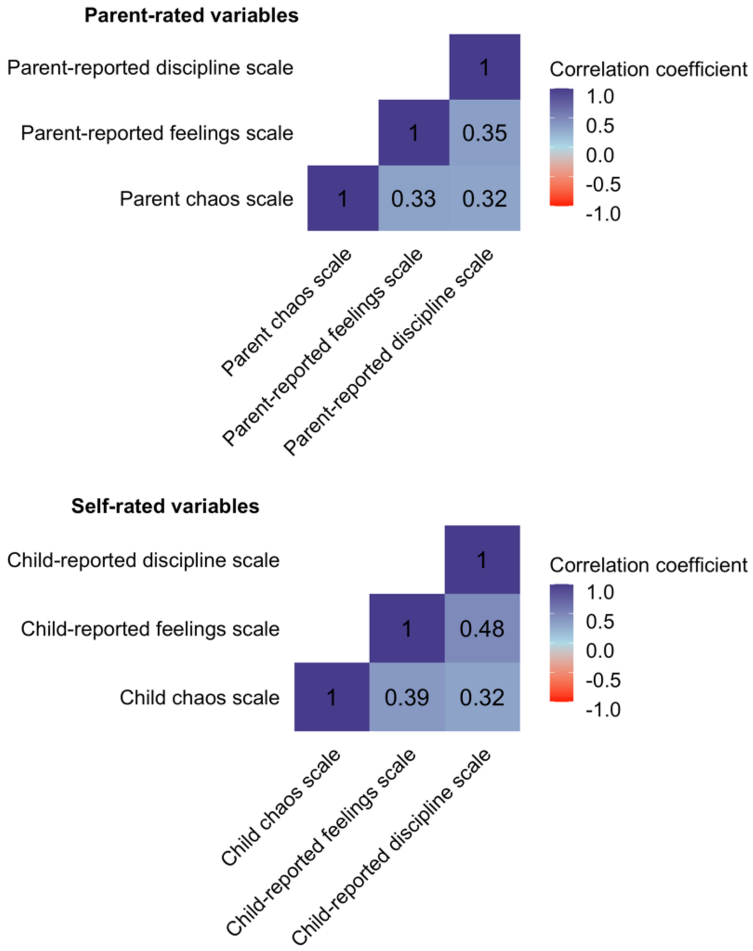

Supplementary Figure 16. Correlations between parent and self-rated environmental variables at age 12.

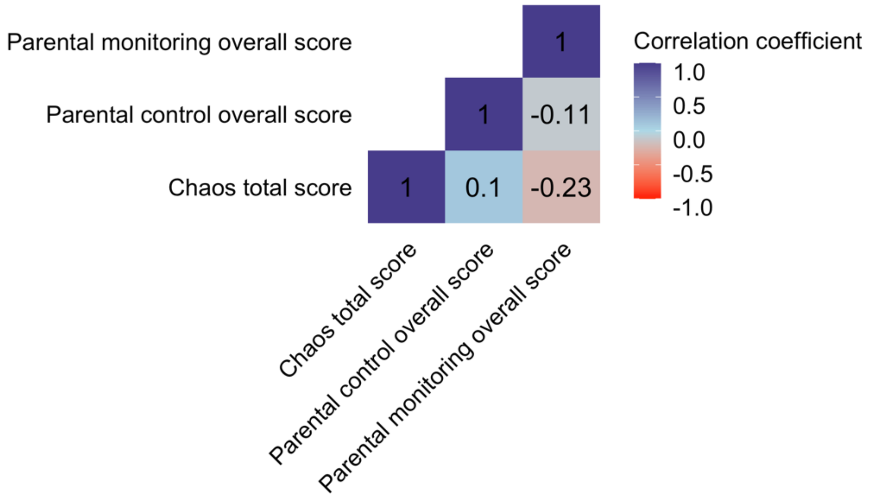

Supplementary Figure 17. Correlations between self-rated environmental variables at age 16.

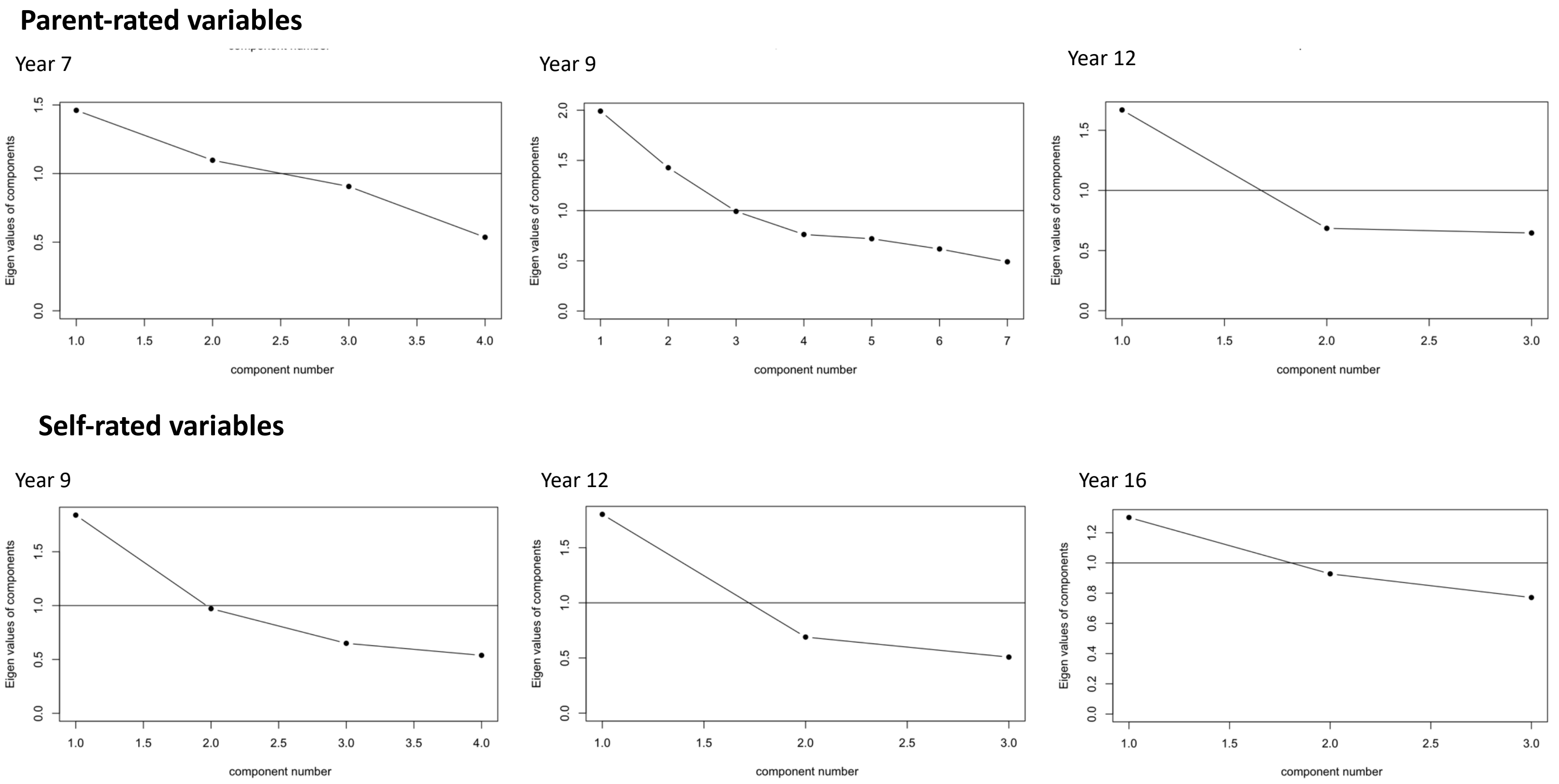

Supplementary Figure 18. Scree plots of parent and self-rated environmental variables at first contact and ages 7, 9, 12 and 16.

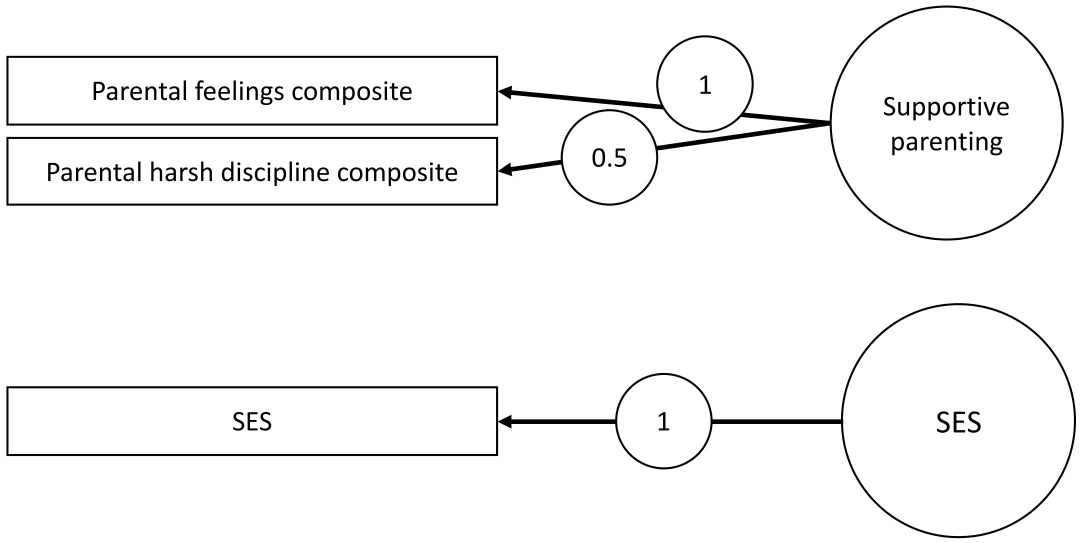

Supplementary Figure 19. Factor structure of parent-rated environmental variables at age 7.

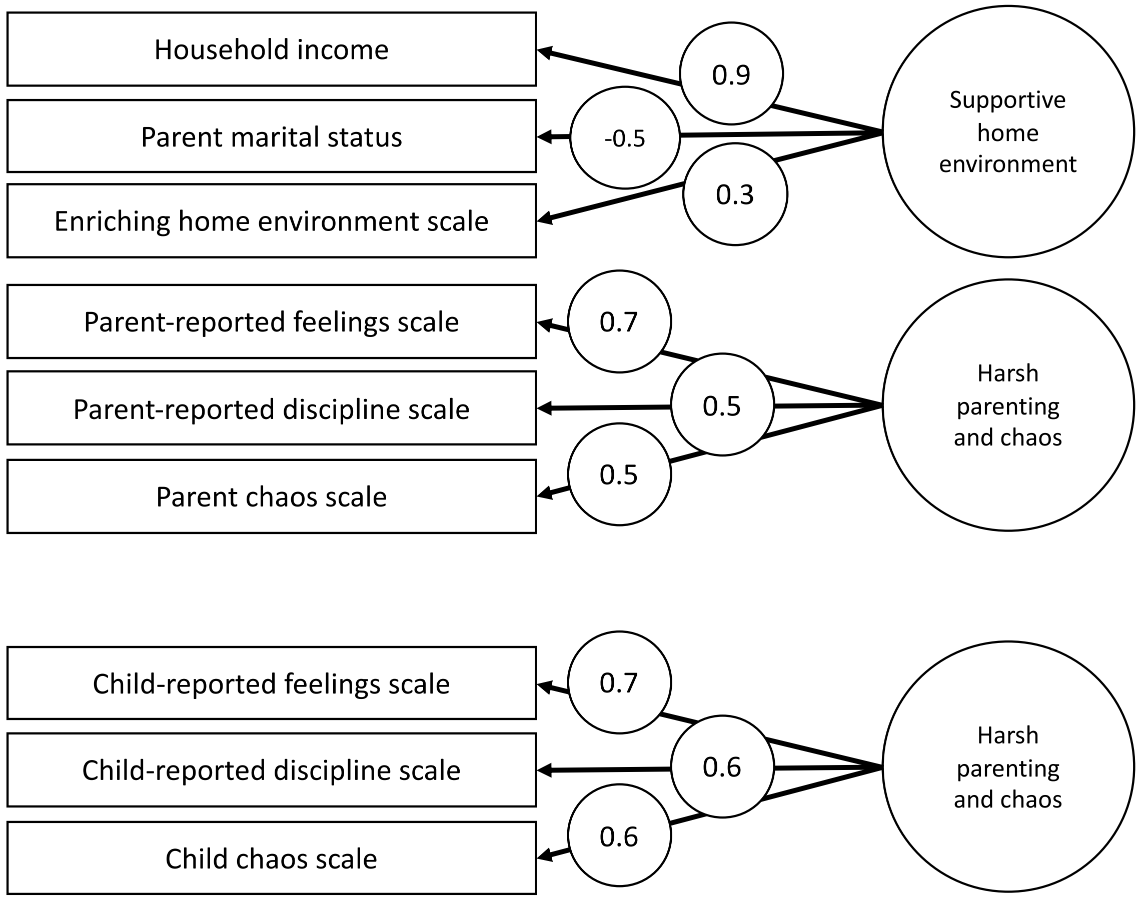

Supplementary Figure 20. Factor structure of parent and self-rated environmental variables at age 9.

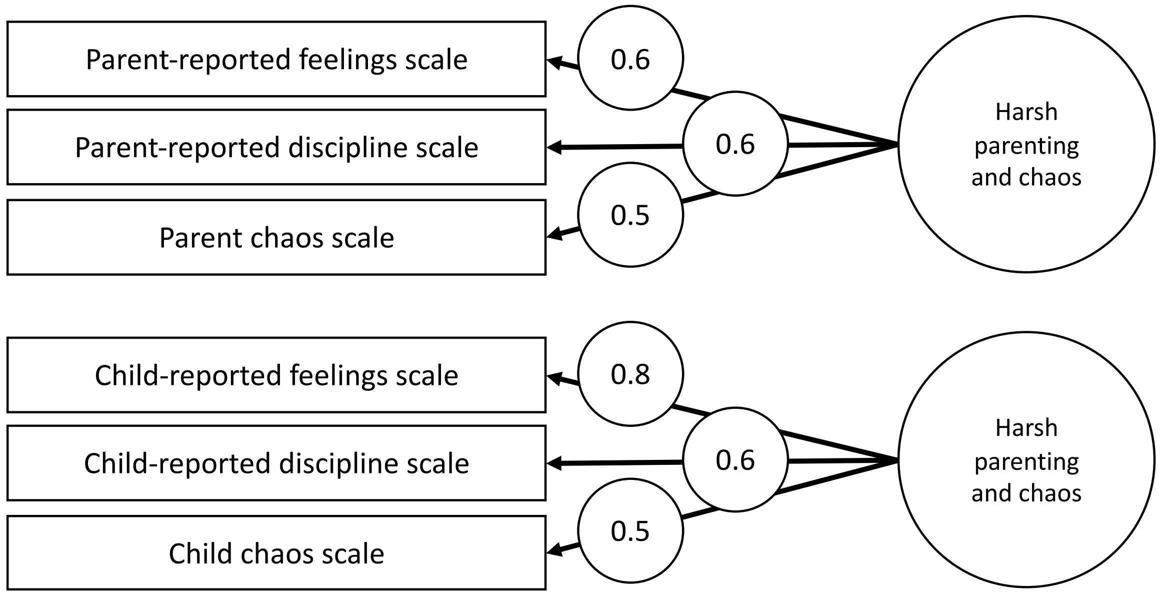

Supplementary Figure 21. Factor structure of parent and self-rated environmental variables at age 12.

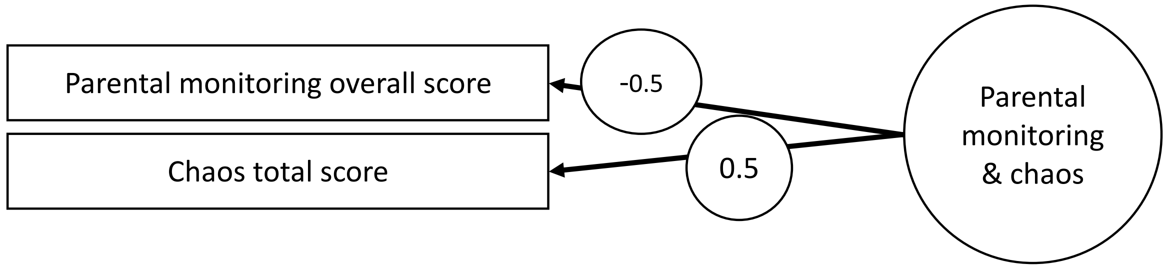

Supplementary Figure 22. Factor structure of self-rated environmental variables at age 16.

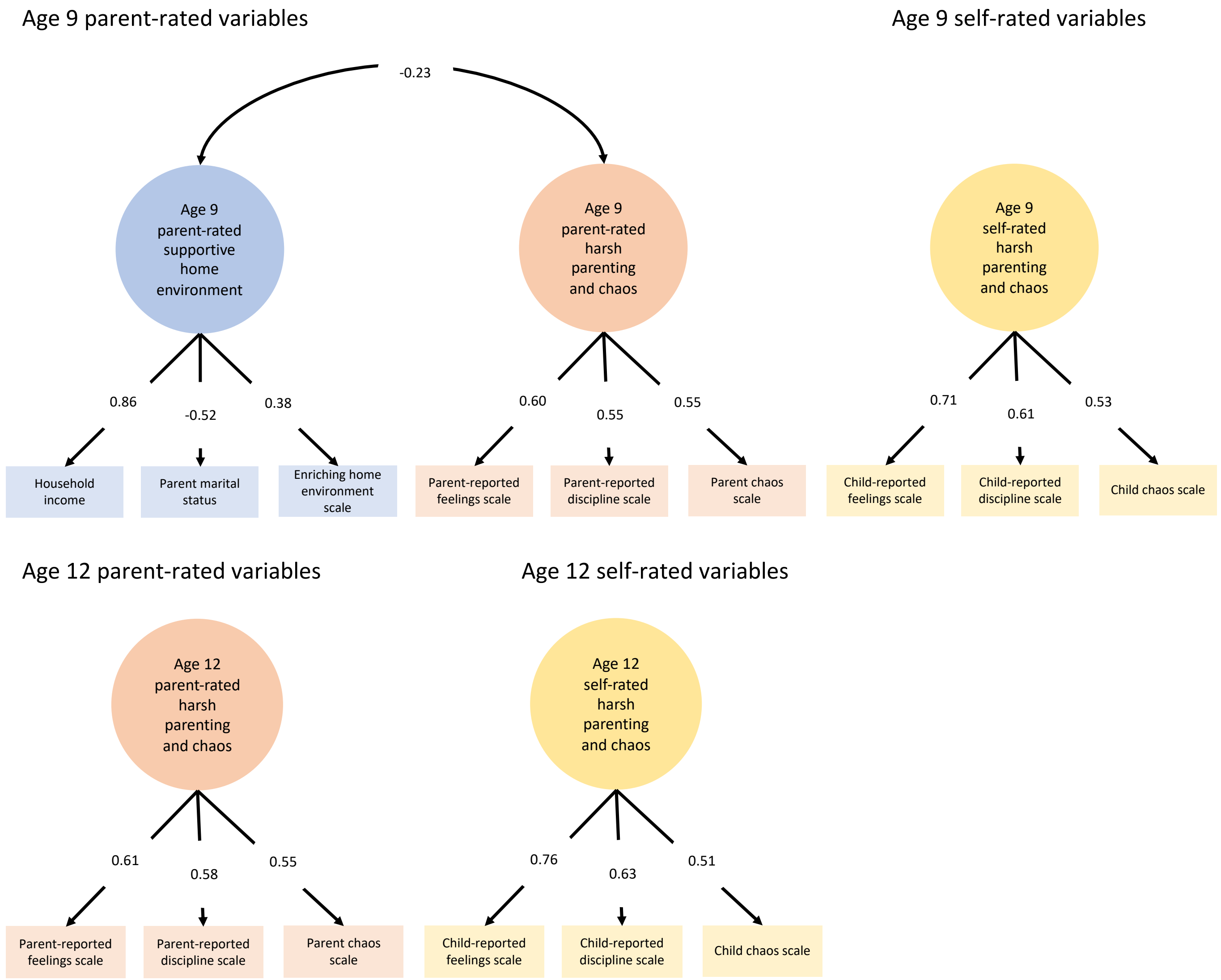

Supplementary Figure 23. CFA models of parent and self-rated latent cross-sectional composites at ages 9 and 12.

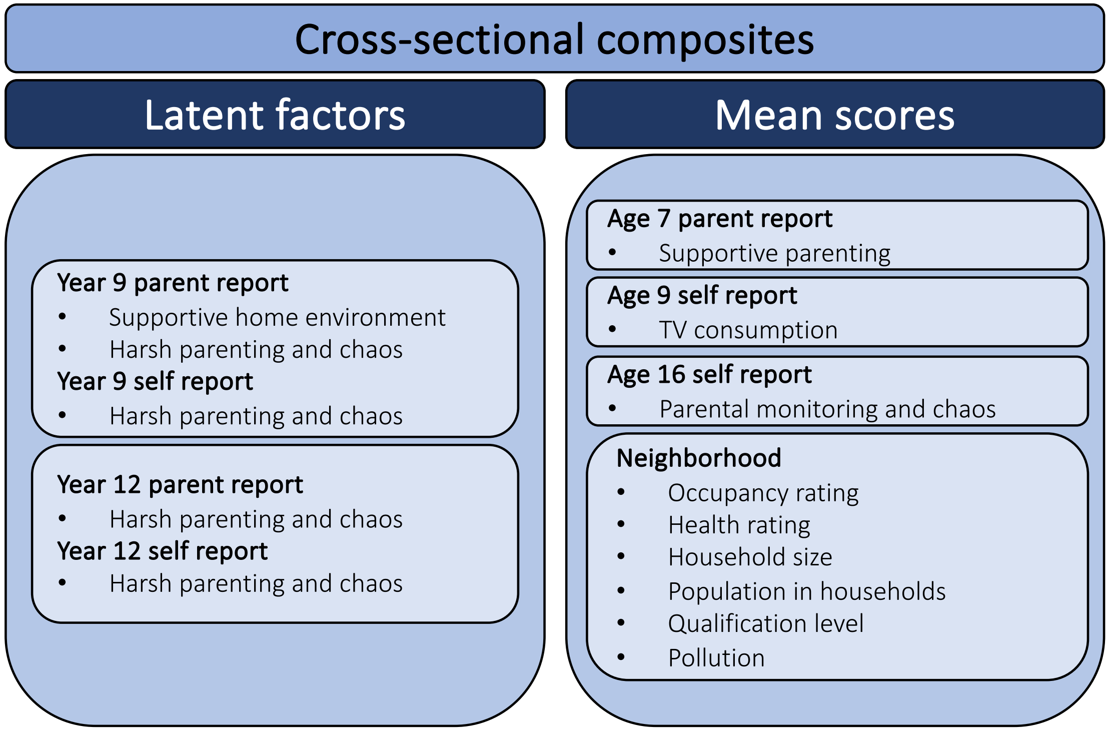

Supplementary Figure 24. Cross-sectional composites.

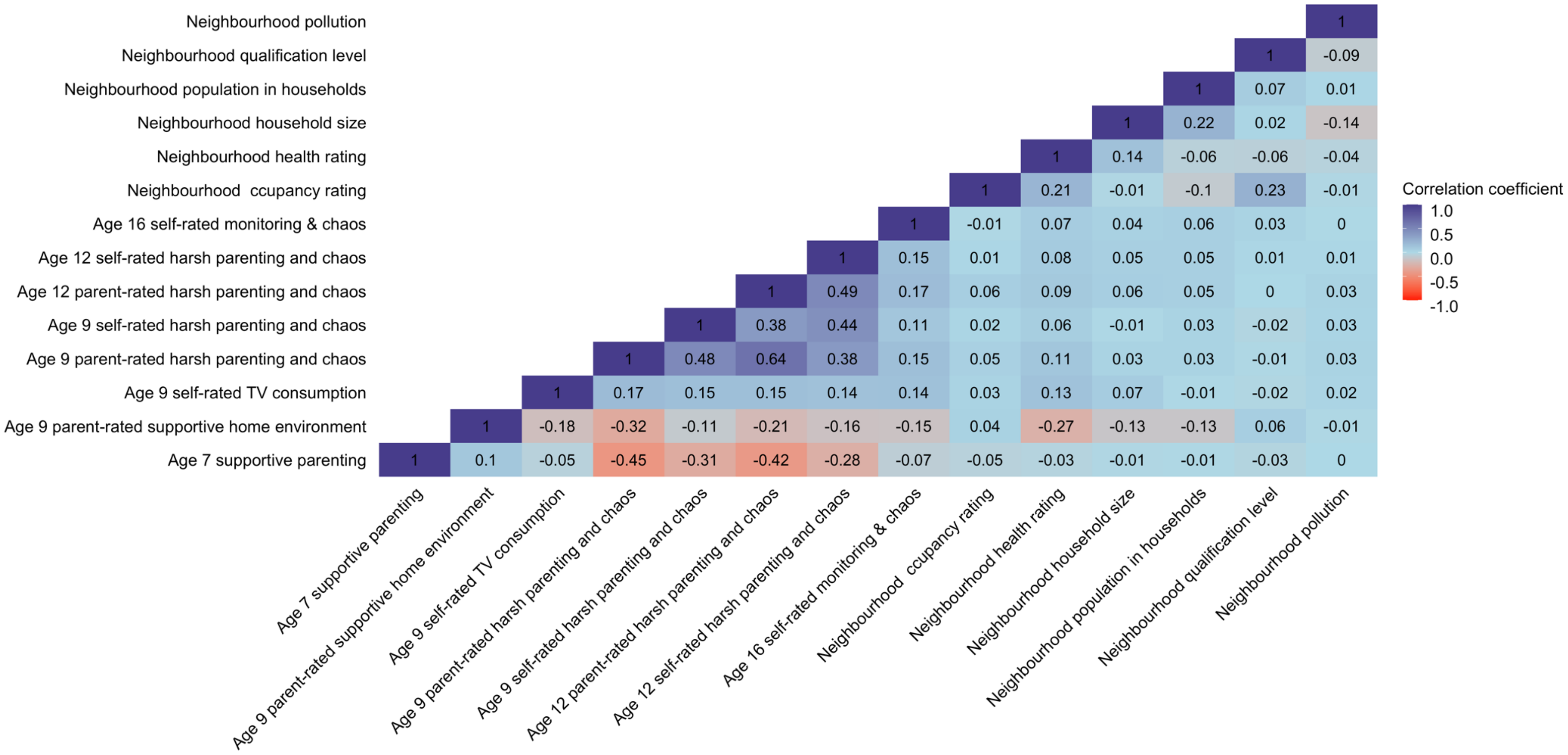

Supplementary Figure 25. Correlations between cross-sectional composites.

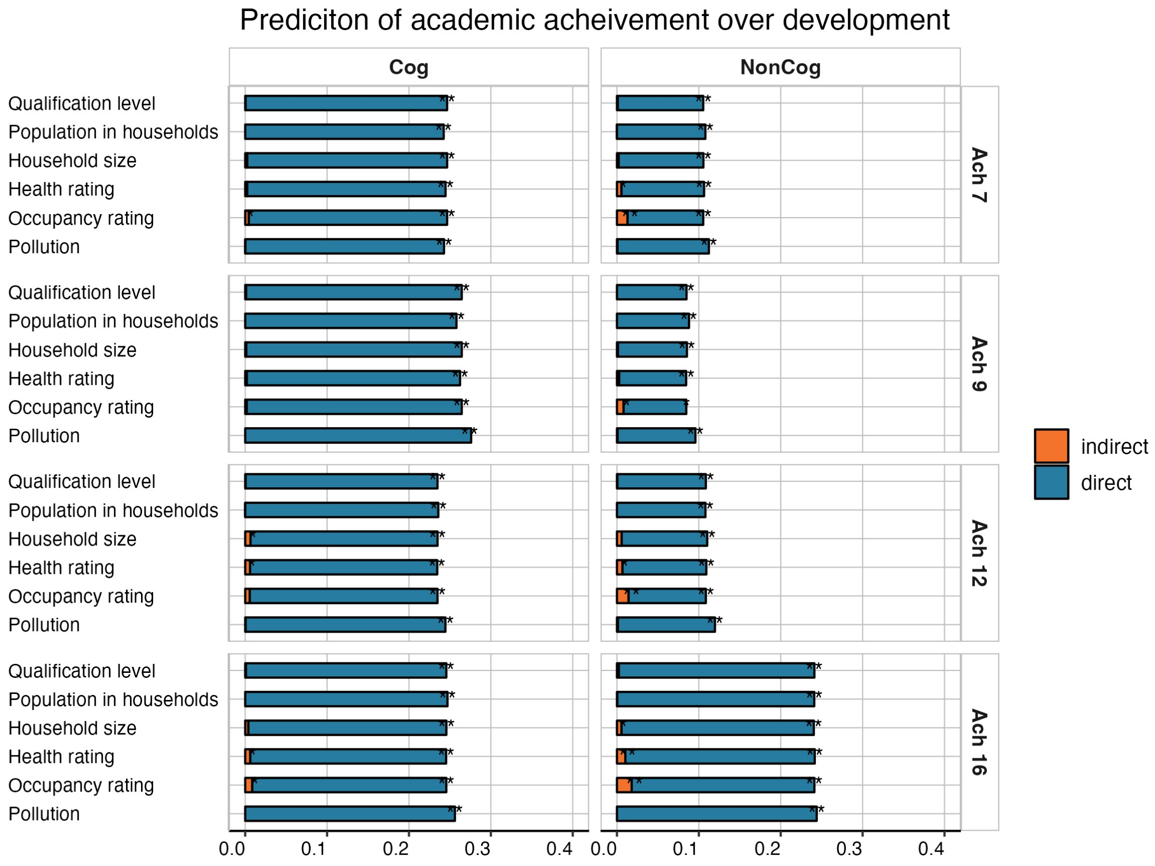

Supplementary Figure 26. Mediation models estimating the indirect effects of the neighbourhood environments on the cognitive (Cog) and noncognitive (NonCog) polygenic score prediction of academic achievement over development.

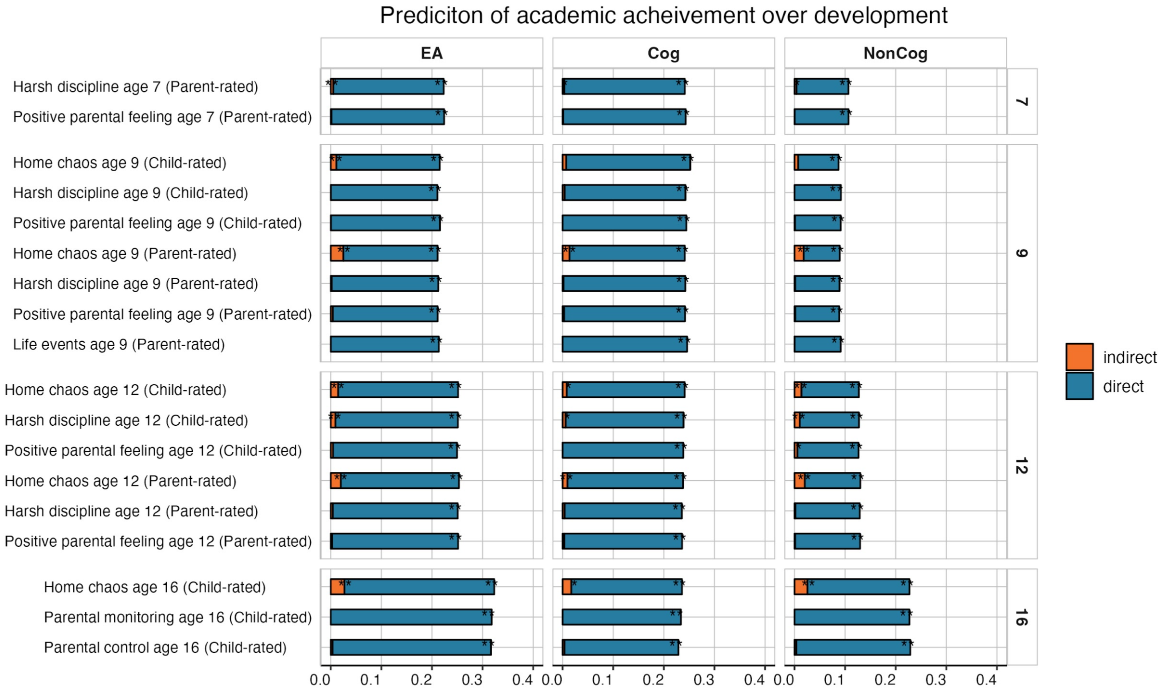

Supplementary Figure 27. Educational attainment, cognitive and noncognitive PGS effects on academic achievement over development mediated by individual measures of the family environment.

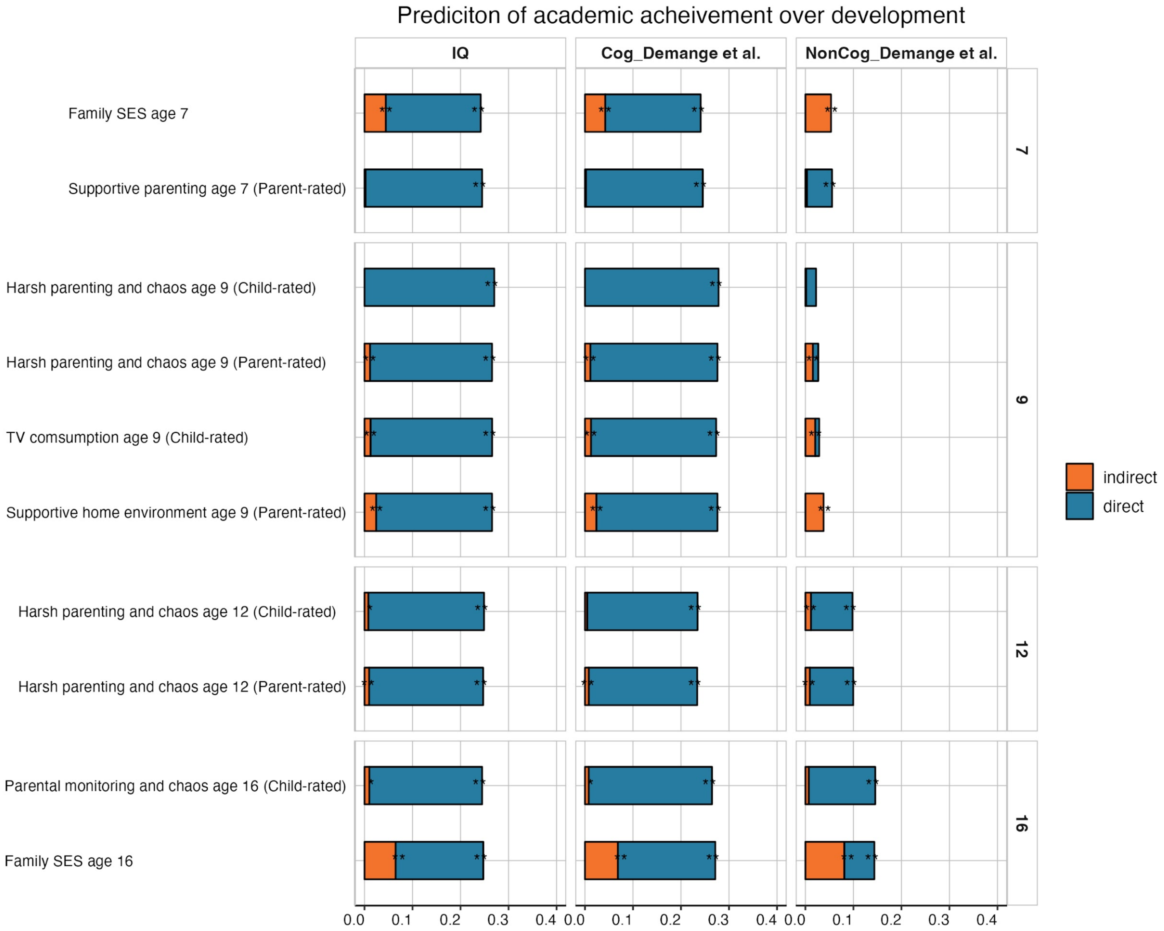

Supplementary Figure 28. Environmental mediation models using other cognitive and noncognitive polygenic scores. Cognitive and noncognitive PGS effects on academic achievementover development mediated by family environmental composites.

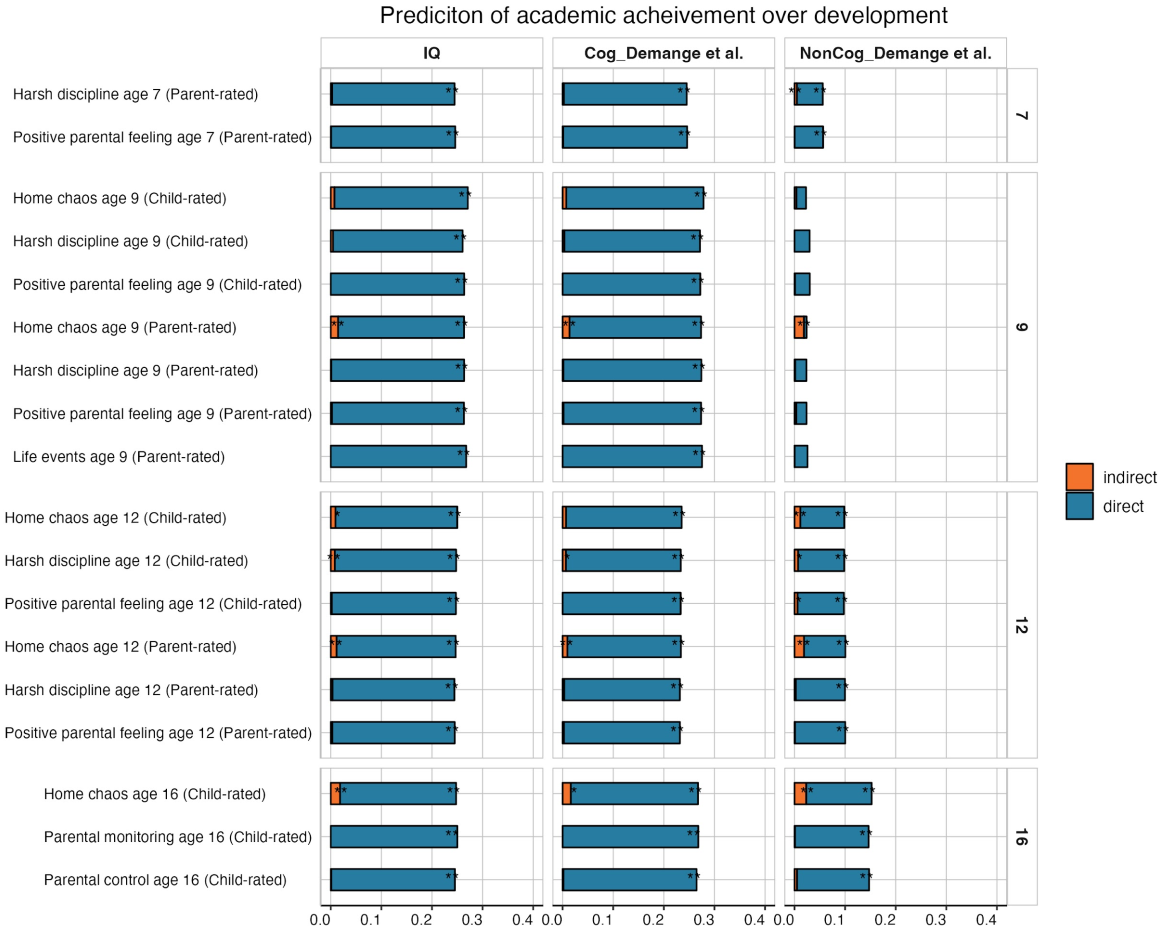

Prediciton of academic acheivement over development
IQ
Cog_Demange et al.
NonCog_Demange et al.
Harsh discipline age 7 (Parent-rated)
Positive parental feeling age 7 (Parent-rated)
Home chaos age 9 (Child-rated)
Harsh discipline age 9 (Child-rated)
Positive parental feeling age 9 (Child-rated)
Home chaos age 9 (Parent-rated)
Harsh discipline age 9 (Parent-rated)
Positive parental feeling age 9 (Parent-rated)
Life events age 9 (Parent-rated)
Home chaos age 12 (Child-rated)
Harsh discipline age 12 (Child-rated)
Positive parental feeling age 12 (Child-rated)
Home chaos age 12 (Parent-rated)
Harsh discipline age 12 (Parent-rated)
Positive parental feeling age 12 (Parent-rated)
Home chaos age 16 (Child-rated)
Parental monitoring age 16 (Child-rated)
Parental control age 16 (Child-rated)
7
9
12
16
0.0 0.1 0.2 0.3 0.4
indirect
direct

Supplementary Figure 29. Environmental mediation models using other cognitive and noncognitive polygenic scores. Cognitive and noncognitive PGS effects on academic achievement over development mediated by individual measures of the family environment.

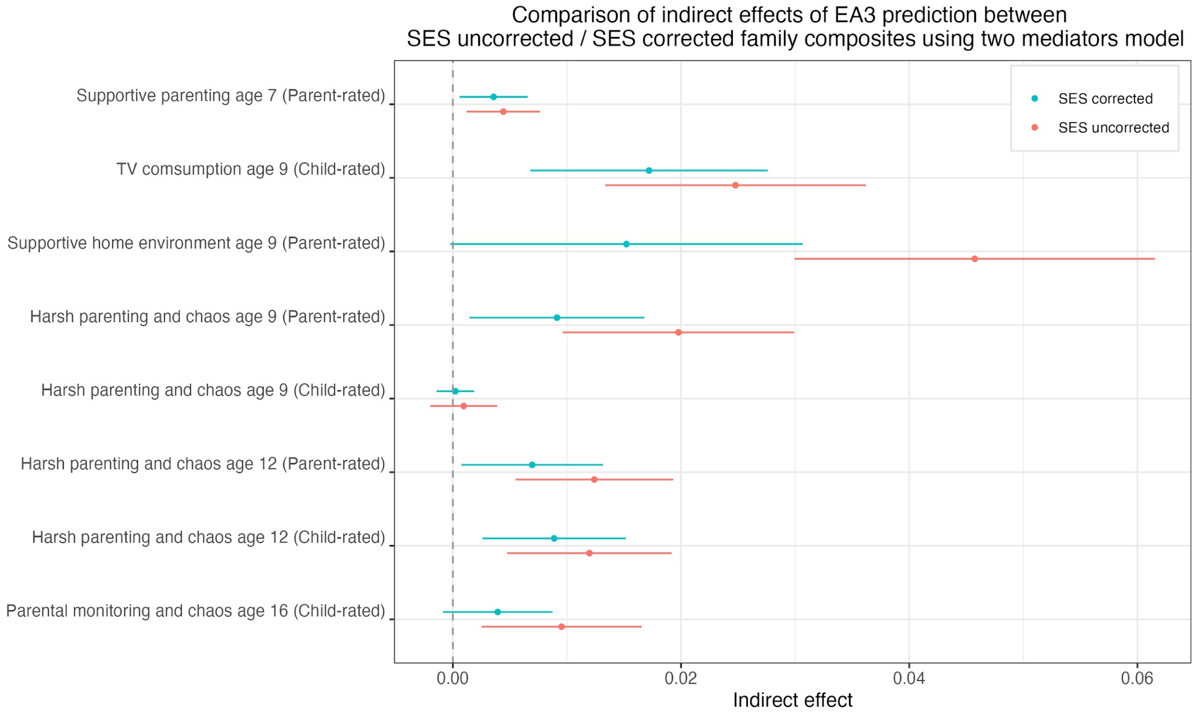

Supplementary Figure 30. Indirect (environmentally mediated) educational attainment PGSeffects on academic achievement before (pink) and after (blue) accounting for SES using two-mediators models.

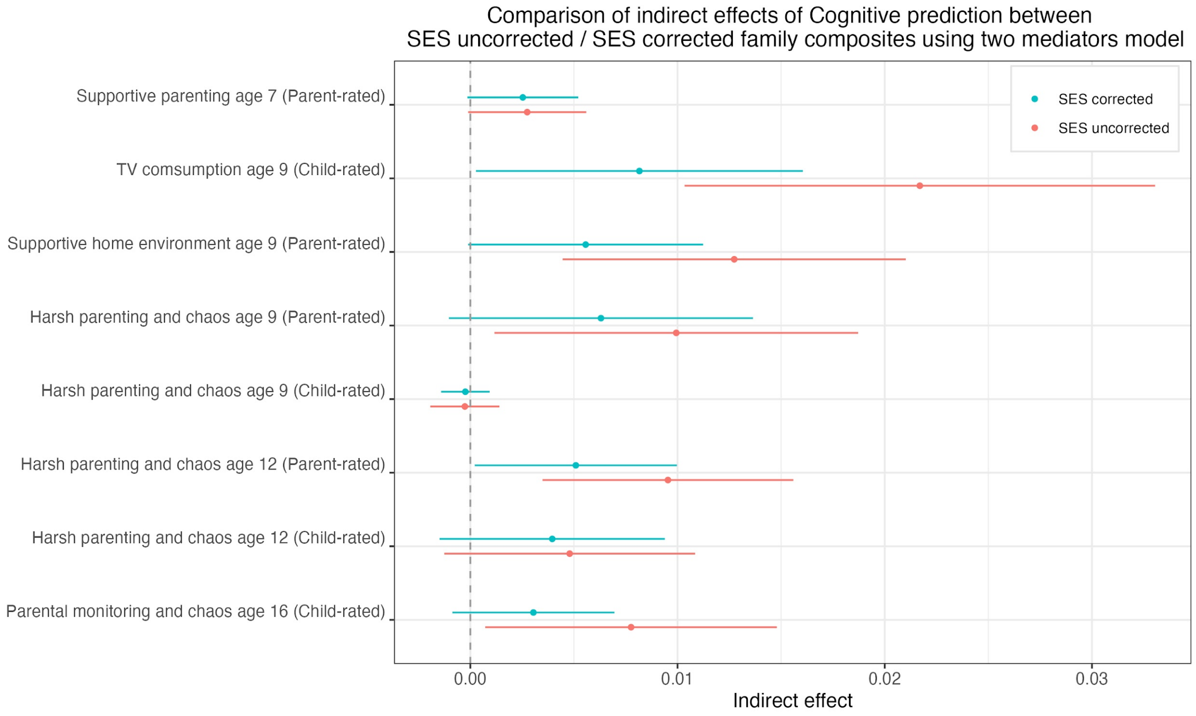

Supplementary Figure 31. Indirect (environmentally mediated) cognitive PGS effects on academic achievement before (pink) and after (blue) accounting for SES using two-mediators models.

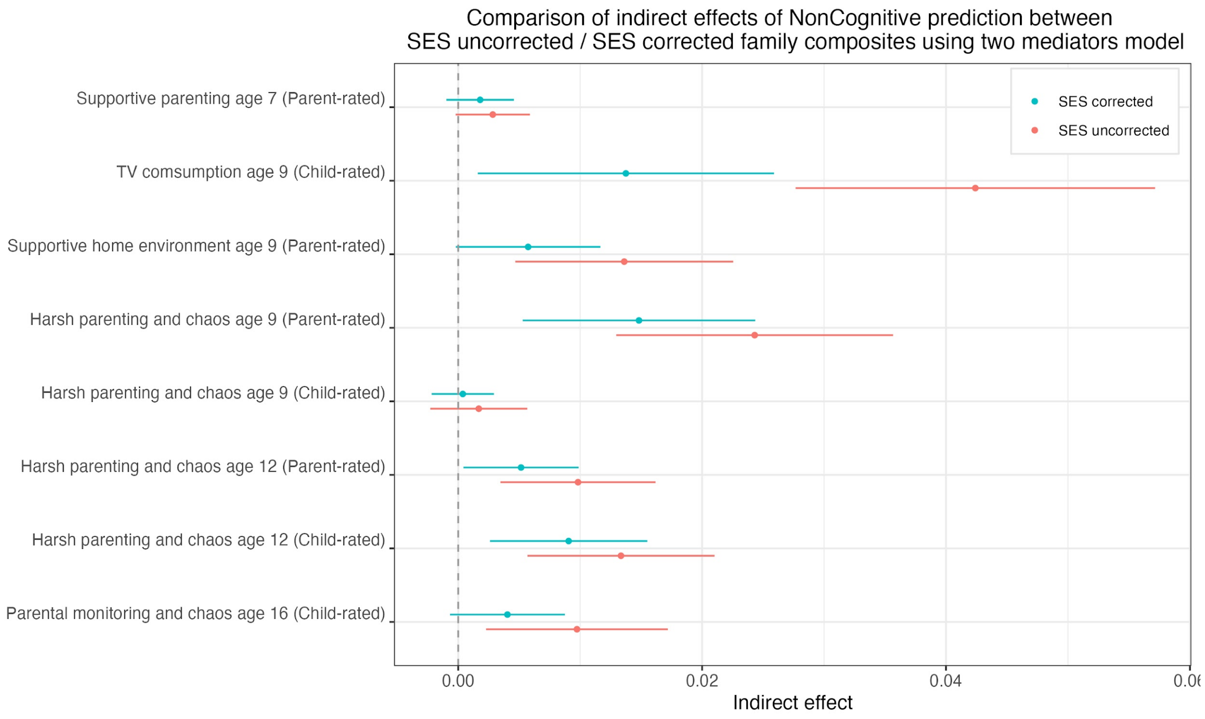

Supplementary Figure 32. Indirect (environmentally mediated) noncognitive PGS effects on academic achievement before (pink) and after (blue) accounting for SES using two-mediators model.

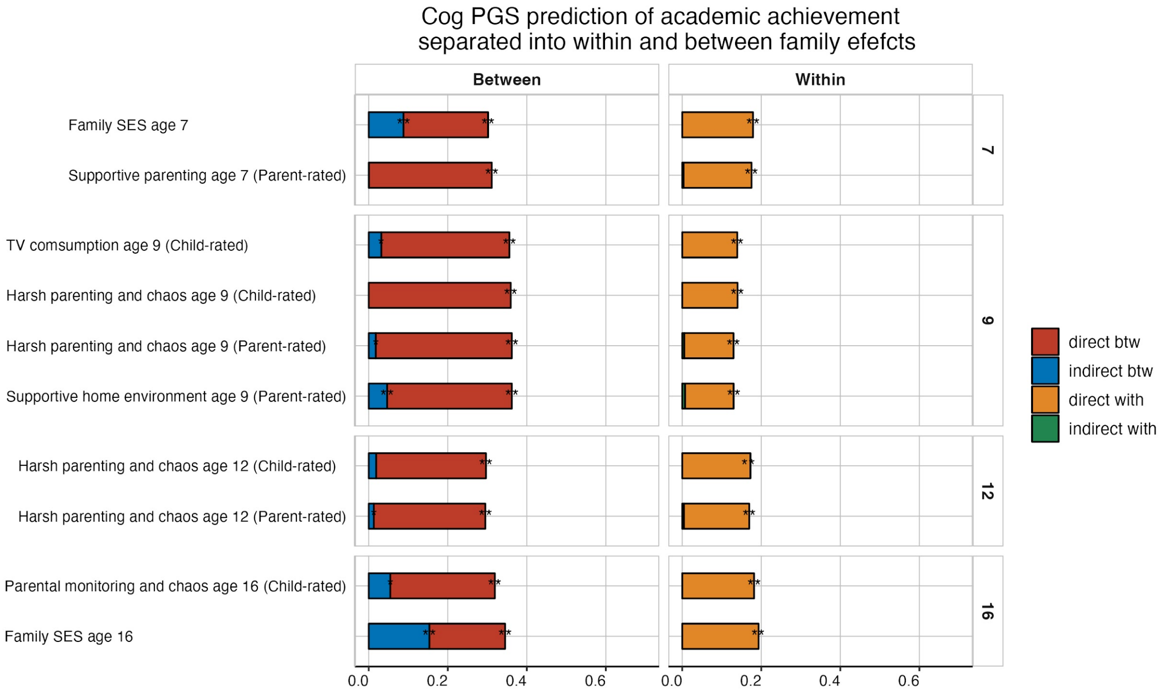

Supplementary Figure 33: Environmentally mediated cognitive PGS effects on academic achievement across development, separated into within and between family effects.

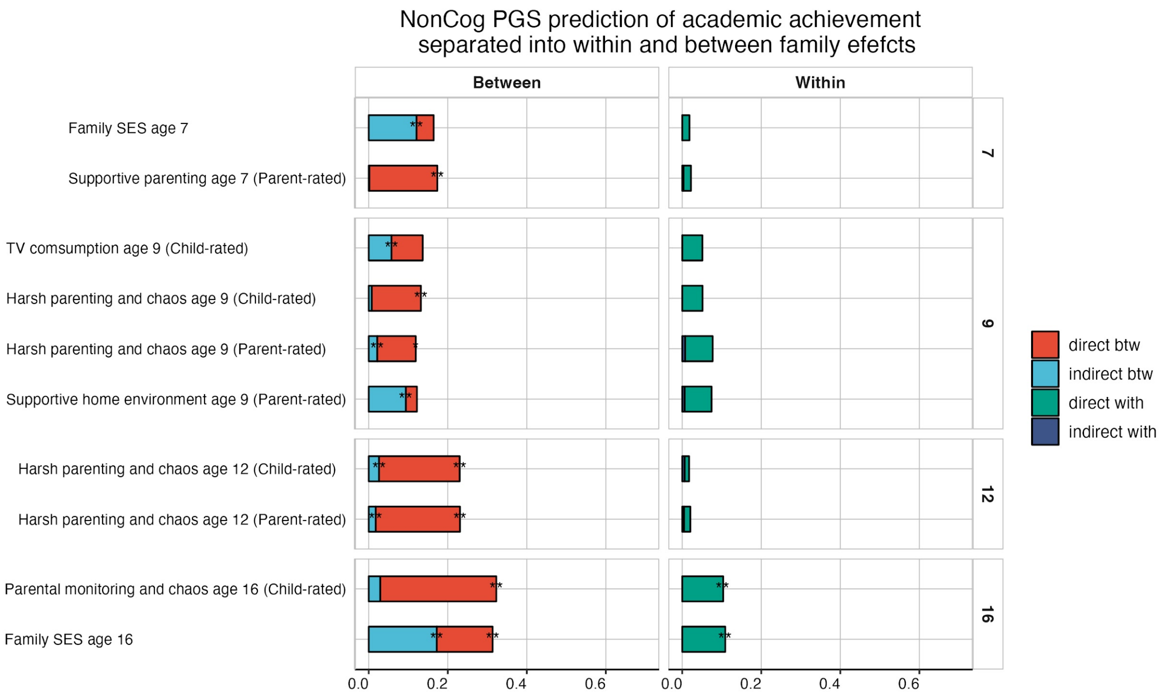

Supplementary Figure 34. Environmentally mediated noncognitive PGS effects on academic achievement across development, separated into within and between family effects.

References:

1. Revelle W, Revelle MW. Package 'psych'. Compr R Arch Netw. 2015;337:338.
2. Team RC. R: A language and environment for statistical computing (R Version 4.0. 3, R Foundation for Statistical Computing, Vienna, Austria, 2020). Google Sch There No Corresp Rec This Ref. 2021;
3. Gunzler D, Chen T, Wu P, Zhang H. Introduction to mediation analysis with structural equation modeling. Shanghai Arch Psychiatry. 2013 Dec;25(6):390–4.
4. Demange PA, Malanchini M, Mallard TT, Biroli P, Cox SR, Grotzinger AD, et al. Investigating the genetic architecture of noncognitive skills using GWAS-by-subtraction. Nat Genet. 2021 Jan;53(1):35–44.
